# A Multidisciplinary, Science-Based Approach to the Economics of Climate Change

**DOI:** 10.3390/ijerph8040985

**Published:** 2011-04-01

**Authors:** Alan Carlin

**Affiliations:** Carlin Economics and Science, Fairfax, VA 22031, USA; E-Mail: carlineconomics@gmail.com

**Keywords:** environmental economics, climate change, economic benefits, costs, multidisciplinary, scientific method

## Abstract

Economic analyses of environmental mitigation and other interdisciplinary public policy issues can be much more useful if they critically examine what other disciplines have to say, insist on using the most relevant observational data and the scientific method, and examine lower cost alternatives to the change proposed. These general principles are illustrated by applying them to the case of climate change mitigation, one of the most interdisciplinary of public policy issues. The analysis shows how use of these principles leads to quite different conclusions than those of most previous such economic analyses, as follows:

- The economic benefits of reducing CO_2_ emissions may be about two orders of magnitude less than those estimated by most economists because the climate sensitivity factor (CSF) is much lower than assumed by the United Nations because feedback is negative rather than positive and the effects of CO_2_ emissions reductions on atmospheric CO_2_ appear to be short rather than long lasting.
- The costs of CO_2_ emissions reductions are very much higher than usually estimated because of technological and implementation problems recently identified.
- Geoengineering such as solar radiation management is a controversial alternative to CO_2_ emissions reductions that offers opportunities to greatly decrease these large costs, change global temperatures with far greater assurance of success, and eliminate the possibility of low probability, high consequence risks of rising temperatures, but has been largely ignored by economists.
- CO_2_ emissions reductions are economically unattractive since the very modest benefits remaining after the corrections for the above effects are quite unlikely to economically justify the much higher costs unless much lower cost geoengineering is used.
- The risk of catastrophic anthropogenic global warming appears to be so low that it is not currently worth doing anything to try to control it, including geoengineering.

## 1. Introduction

With the development of stated preference, travel cost, benefits transfer, and other techniques that do not require a full understanding of the physical science aspects of valuing environmental benefits, environmental economics has tended to pay less attention to these aspects. But this tendency has brought some problems as well as some benefits for environmental economists. These problems are particularly important in the case of economic analyses of multidisciplinary problems such as climate change. Economic analyses of environmental mitigation and other public policy issues can be much more useful if they critically examine what other disciplines have to say, insist on using the most relevant observational data and the scientific method, and examine lower cost alternatives to the change proposed. These general principles are illustrated by applying them to the case of climate change mitigation, one of the most interdisciplinary of public policy issues, and showing how they lead to quite different conclusions than those of most previous such economic analyses. This seems likely to be the case for other interdisciplinary public policy issues besides climate change.

There is little doubt that proposals to mitigate the threat of climate change, sometimes referred to as catastrophic anthropogenic climate change (CAGW) or global warming, has presented environmental economics with its most important challenge to date in terms of providing useful advice on what if anything should be done concerning what is perhaps the major environmental public policy issue of the last decade or more. The economic implications of some prominent proposals to control climate change are large. There is currently probably no public policy issue with greater economic impact on which environmental economists might have particular expertise and therefore something useful to say. The purpose of this article is to assess whether these efforts would be more effective if economists used these principles.

This article was initially motivated by personal discussions with two of the principal economic analysts on this subject concerning the validity of the physical science inputs that they had used. One said that he had never examined the physical science inputs he used from the United Nations (UN) Intergovernmental Panel on Climate Change (IPCC) efforts since he assumed that they were valid. The other said that he believed there should be a division of effort and that since his expertise was not in the physical sciences this should be left to experts in this field, which he felt included the UN IPCC.

### 1.1. Use of Most Relevant Observational Data and the Scientific Method as the Basis for Determining Effects

This article will assume, I think reasonably, that the criterion for use of an assumption or hypothesis is whether it is supported by relevant observational tests or by the use of the scientific method. If the assumption or hypothesis is not so supported, this means that it is not supported by real world data since the role of relevant observational tests and the scientific method is to make sure that assumptions and hypotheses actually correspond with reality. Under this approach, assertions of alleged facts, model claims, and questionable arguments (such as that the authors cannot think of any alternative explanations for their conclusions) are not considered. This differs substantially from much previous physical science and economic research on climate change, but for good reason.

It is important that environmental economics be based on valid science and not conjecture or religious belief if it is to be a reliable guide for policy making. There is no other way to lay a firm foundation for environmental economics so it corresponds to the real world rather than the current fashions of the day.

According to my definition of valid science in Section 1.2, it is important for economists to try to clearly identify those physical science conclusions they are using that are supported by specific observational tests and the application of the scientific method and which are not. Those not supported by observable real world data or the scientific method are best identified as hypothetical calculations in my view so as not to be confused with scientific conclusions. Although such hypothetical analyses can play an important role in some cases, they need to be carefully distinguished from conclusions of more general applicability. This includes conclusions endorsed by national academies of science, scientific societies, peer reviewers, model outputs, or governmental organizations. Hypothetical effects can be often be usefully in analyzed, of course, but it is vital if economists are to present credible advice to policy makers that any conclusions reached using such assumptions and hypotheses be clearly labeled as being not verified scientifically so that the user keeps these qualifications in mind when using the conclusions resulting from their use. A similar approach is needed in those cases where there is insufficient evidence to determine whether assumptions or hypotheses represent valid science.

### 1.2. Why Science Should Be Based on Relevant Observational Data and the Scientific Method

It is very important to understand what valid science is. Valid science as used here is the result of using the most relevant observational data and the scientific method to understand the world and its inhabitants. The most relevant goal needs to take into account the reliability of the observational data. The essential elements of the scientific method are characterizing the subject of inquiry, generating a theoretical, hypothetical explanation for the characterizations, making predictions based on the hypothesis, and finally experimentally determining the validity of these predictions by comparisons between the predictions and real world data. The determination of validity needs to be reproducible and independently verifiable. The assumptions and hypotheses being tested throughout this paper are those found in the various United Nations (UN) Intergovernmental Panel of Climate Change (IPCC) reports concerning climate change, reasonable reformulations of those found there, or interpretations of them by economists preparing benefit estimates.

Valid science is not writing a description of the world or the opinions of world authorities on a particular subject. Valid science is not a statement of belief by scientific organizations. The question is not what someone believes but whether what he or she believes corresponds to the most relevant observational data and the scientific method. It is important to note that science evolves over time as new discoveries are made and new hypotheses are formulated and discarded. Only continuing research can insure that important new research results become available. Richard Feynman [1] expressed this as follows:

“In general, we look for a new law by the following process. First, we guess it. Then we compute the consequences of the guess to see what would be implied if this law that we guessed is right. Then we compare the result of the computation to nature, with experiment or experience; compare it directly with observation to see if it works. If it disagrees with experiment it is wrong. It’s that simple statement that is the key to science. It does not make any difference how beautiful your guess is. It does not make any difference how smart you are, who made the guess, or what his name is—if it disagrees with experiment (observation) it is wrong.”

According to the scientific method, a scientific hypothesis should be tested by comparing real world data with the implications of the hypothesis. This is how Albert Einstein’s ideas on relativity were ultimately accepted. Many scientists doubted his hypothesis, and repeatedly proposed real world tests of it. But each test confirmed its validity or was shown to be based on faulty data or analysis. After a number of these tests, the opposition conceded that his hypothesis was valid. This process is described in Crelinsten [2]. The same process is needed in the case of climate change hypotheses.

There are alternative approaches that have been proposed to the use of the scientific method for determining scientific validity. An example is that proposed by Armstrong and Green [3]. Their broader approach includes many of the attributes of using the scientific method outlined above, and reaches the same conclusion as this study does as to the validity (or lack thereof) of much current research on climate change, but places somewhat less emphasis on the use of observational data and the validity of hypotheses.

#### 1.2.1. Why Computer Models Do Not Demonstrate Scientific Validity

CAGW supporters often claim that the results of climate models validate their conclusions scientifically. Although these climate general circulation models (GCMs) have many uses in scientific inquiry, they do not prove or disprove scientific validity. Models show what the model builders believe is the way some physical system, in this case climate, works. But all that they represent (at best) is the model builders’ beliefs and judgments. Models produce scenarios, not forecasts [3], and neither prove nor disprove scientific hypotheses nor show what will happen in the future. The only way to do this is by comparison of particular scientific hypotheses with real world data other than past temperature data, which the models have usually been modified to emulate. The particular climate models used by the UN IPCC appear to be even more questionable than many since they do not even do a good job of hindsight [4], let alone foresight.

#### 1.2.2. Uncertainties in the Scientific Data Used for Rejecting a Hypothesis

Although the principle of using the most relevant observational data and the scientific method as outlined above for deciding what is and is not valid science and hence what should and should not be used in economic analyses appears inescapable, its application to particular issues is often more difficult. There will inevitably be questions about whether the correct comparisons have been made between the hypotheses to be tested and the real world data used to test them, or whether the best data have been used to make the comparisons, or whether there even is valid, unmanipulated, and adequate quality data that can reasonably be used for such comparisons, or whether the most recent research results have been used, or what constitutes research results that should be used.

In my view there is no such thing as settled science because settled science is not science. After a scientific hypothesis has undergone many comparisons with real world data it may gradually assume the status of a physical law. But short of such laws, and in some cases even in their case, science only advances by constantly questioning all aspects of any hypothesis. So although I will attempt to use what I consider to be the most valid science available to me at the time this is written, and am willing to defend my judgments as such, future research may result in either further evidence confirming my judgments or even refuting them. If the risk of such changes is high and the cost of undertaking a policy change is high, the best approach is normally to do nothing until either the science becomes more settled or the costs come down. It should be the responsibility of those proposing a public policy change to make the case in this respect, not those opposed to making the change.

An important footnote here is that valid science as used here is determined by whether it is based on the most relevant observational tests and the scientific method, not by whether it has or has not appeared in a peer-reviewed journal. Peer-review may play an important role in determining which articles are published, but does not guarantee that the assumptions and hypotheses used represent valid science since the primary requirement for successful peer review is that other experts agree with the author. Particularly if enough experts agree on one particular viewpoint or if experts who do not agree are excluded from the peer review, peer-review does not guarantee scientific validity, the primary criterion used in this article.

Unfortunately, the only useful way to deal with the inevitable application uncertainties is on a case-by-case basis. General rules such as only peer-reviewed, published research should be judged as valid may simplify decision-making, but impose substantial costs in terms of delays and the discouragement of new ideas, and in no way guarantee that all such research represents valid science. In the end, reasonable judgments have to be made. Much as economists might like to avoid these “scientific” issues or may even feel unsuited to judge them, economists run the risk of grossly misleading their readers if they fail to deal with the major scientific (as well as economic) uncertainties and their economic implications. Where these uncertainties are very large, a reasonable conclusion may be that detailed benefit-cost analysis cannot play a useful role. But in most cases a careful analysis of the major scientific uncertainties will avoid the most serious risks of misinforming readers. At the very least, the approach used here provides a framework for deciding which physical science assumptions and hypotheses to accept and which to reject. This general approach has too often been lacking in discussions of climate change issues by economists as well as non-economists. In all too many cases these discussions have centered on prejudicial denigration of what the opposition is saying or the motives of the individuals saying it. It is only by using the most relevant observationally derived data and applying the scientific method that progress can be made on this and many other environmental and public policy interdisciplinary issues involving the physical sciences. The discussion should be centered on what the most relevant evidence shows, not who is making the argument.

This paper can be viewed primarily as an attempt to apply the most relevant observational data and the scientific method to determine whether a few of the most economically critical assumptions and hypotheses are scientifically valid in the climate change case and what the economic implications of using other, more valid assumptions and hypotheses would be. Secondarily, it examines what the effects of ignoring lower cost mitigation alternatives are. As such, it has important methodological implications even if readers should disagree with the judgments reached as to the validity of some of the physical science assumptions and hypotheses central to the debate or with the validity of using lower cost alternatives. Some may argue that physical science decisions should be left to physical scientists and that economists should not get involved in the physical science aspects of policy choices they are evaluating; I argue, on the contrary, that there is no escape from making such judgments since using the scientific judgments of one side in a scientific argument assumes that that side is valid and the opposition side is invalid. The result is that the casual reader may not even realize that there is a difference of opinion and what effects that difference has on the economic analysis. All this will be illustrated by the case of climate change mitigation. Even if the reader should disagree with the particular judgments made in this article concerning the scientific validity or invalidity of various assumptions and hypotheses, I believe I have at least demonstrated the importance of explicitly explaining the effects of the major scientific uncertainties in any economic analysis with major interdisciplinary characteristics such as climate change mitigation. At the very least, I believe this study shows that the outcomes of economic analyses of the climate change mitigation are critically sensitive to a number of scientific judgments which need to be very carefully examined for scientific validity.

#### 1.2.3. Some Assumptions

This article will assume that most of the economists who have attempted to value the economic benefits of climate change remediation have directly or indirectly tried to use the then most recent version of the IPCC assessment reports as their guide to the science. Some may have been more or less successful in doing so, of course. But in order to make some more general points with regard to how economic analyses of such hypotheses might best be carried out, there is no other easy way to do so other than by making this assumption; further, this seems to be the underlying unifying assumption of all the previous analyses discussed in this article. Thus by questioning a particular aspect of the IPCC’s analyses, it is assumed that all of the economic analyses are questioned in this respect. Each economic analysis is different in what it computes the benefits will be, what discount rate is used, *etc.*, but most appear to strive towards using IPCC science in determining what the economic benefits of climate change mitigation might be as it was known at the time the analysis was done.

### 1.3. Why Economists Also Have a Responsibility to Consider the Lowest Cost Alternative

In doing benefit-cost analysis economists can usually select from a variety of alternative ways to achieve the benefits being valued. If they select a higher cost alternative for their analysis, they are putting the action being valued at a disadvantage relative to using the lowest cost alternative. So although the action might be economically efficient using the lowest cost alternative, it might not be using the higher cost alternative. In any case it would be more efficient using the lowest cost alternative. I will take the viewpoint that economists would be able to make a more useful policy contribution by pointing out whether lower cost alternatives exist so that users can determine what the meaning of the conclusions reached might be in terms of all the options available. Some of these alternatives may have unquantifiable disadvantages compared to higher cost alternatives, but this needs to be pointed out so that policy analysis users can select the best possible option rather than simply ignoring or not discussing these lower cost alternatives as too often happens, particularly in the case of climate change.

### 1.4. Outline of the Article

Section 2 will discuss what the economic benefits are of the major solution offered by CAGW proponents for mitigating climate change, namely reducing emissions of greenhouse gases (GHGs) into the air as a result of human activities. Section 3 will discuss several major problems with previous determinations of economic costs. Section 4 discusses how these changes affect benefit-cost analyses of the problem. Section 5 will provide selected comparisons between the conclusions reached in this paper with those of other economists. Section 6 will summarize the major conclusions from the paper.

## 2. The Economic Benefits of Climate Change Mitigation

### 2.1. The Background

Beginning in the 1980s, the United Nations attempted to determine the extent to which global warming may be due to human activities in a series of reports prepared by its IPCC. The most recent of these was issued in 2007 and is usually referred to as the AR4 report [5]. It is important to note that the IPCC’s charge is not to determine what causes global warming but rather whether it results from human activities. They have orchestrated worldwide concern about global warming with strong support from many Western governments, some academics, and most environmental organizations. They concluded that this warming could be mitigated by reducing human emissions of greenhouse gases, particularly carbon dioxide (CO_2_). One of the immediate questions was what the economic benefits of such reductions might be. There have been numerous attempts to determine this by economists, ranging from the technically sophisticated to the more easily understood by the public (see Section 5 below for a list of many of these).

In all major cases I know of those preparing the benefit estimates assumed that the IPCC conclusions concerning the effects of decreasing carbon dioxide and other greenhouse gas emissions were substantially correct and accurate. It is not clear why they all assumed this, however, since as will be shown for particular instances later in this section, key conclusions reached by the IPCC are not supported by the most relevant observational tests and the scientific method (see Section 1.2 above for a discussion of the scientific method and its role in determining what is valid science), and therefore have no standing as science despite numerous statements to the contrary by CAGW supporters. Idso and Singer [6] have compiled an extensive listing of many of these problems.

The IPCC’s timing was excellent since in fact Earth appears to have been in a minor periodic warming period according to ground-based measurements. This minor warming started in the late 1970s and may have peaked in 1998, and followed a much longer standing even more gradual warming that has taken place since the end of the Little Ice Age. A gross generalization is that the IPCC models effectively hypothesized that this minor periodic warming would continue during the 21st century even though similar periodic warming and cooling has been going on since at least the late 19th century and probably for much longer. Although it is not clear whether humans as a whole would on balance be better or worse off as a result of the presumed rise in global temperatures hypothesized by the IPCC, the CAGW promoters managed to sell a major scare to a surprising number of people and government officials through a variety of means. Their views have even been endorsed by most of the national academies of science around the world. Major Western European nations and the European Union enacted significant legislation that would reduce the global warming, or so it was claimed. The United States House of Representatives passed related legislation [7] in 2009, but it was not passed by the Senate and died at the end of the 111th Congress in 2010.

The various UN IPCC reports broadly argue that the authors cannot think of any reasonable source of global warming other than the increasing level of some GHGs, so that must be the cause. There are a few exceptions, such as large volcanic eruptions, which they agree may influence global temperatures, but they do not believe that the warming can be attributed to most other natural causes such as solar or cloud cover variability, which they view as being of minor or little importance in influencing climate.

A much more logical and plausible set of CAGW hypotheses might be as follows:

Hypothesis 1: Anthropogenic releases of CO_2_ are the primary cause of increases in atmospheric CO_2_.
Hypothesis 2: Increases in atmospheric CO_2_ levels interact with the major greenhouse gas, water vapor, to create a large positive feedback capable of creating catastrophic global warming.

If these two hypotheses were correct, then it might follow that:

Hypothesis 3: Anthropogenic GHG emissions, particularly CO_2_, will result in CAGW.

This appears to be the primary hypothesis offered by climate change mitigation promoters. Unfortunately, there is no easy way to determine the validity of this statement with current knowledge other than to examine hypotheses 1 and 2 separately. But a related but still important hypothesis that can be examined is the following:

Hypothesis 3a: Changes in global temperatures are primarily influenced by rising levels of GHGs other than water vapor in the atmosphere.

Most of the remainder of this section will discuss these hypotheses using a variety of sources. The emphasis will be on a few of the scientific issues, which have a major influence on the economic analysis of climate mitigation since economic analysis is the focus of this paper. There are many scientific issues that this article will not discuss because of the time and space that would be required. It is possible, of course, that some of these other issues, if analyzed in the same depth, would result in offsetting the effects of those discussed. Given the history of the UN IPCC, however, which has consistently emphasized the adverse effects of increasing CO_2_ in the atmosphere, this possibility is not very likely.

### 2.2. Hypothesis 1: Anthropogenic Releases of CO_2_ Are the Primary Cause of Increases in Atmospheric CO_2_

There have been slow but fairly steady increases in CO_2_ levels in the atmosphere since measurements began on Mauna Loa in Hawaii in the mid-1950s based on measurements there. Some have argued that these increases are primarily a result of emissions from burning fossil fuels; others believe it is due to natural causes. The IPCC claims that 21 percent of atmospheric CO_2_ is from burning fossil fuels [8], and that CO_2_ may remain in the atmosphere for a long period, with a “rough indication” of 50 to 250 years, as discussed in the next paragraph. The atmospheric residence time (or RT) is less than clearly stated in the various IPCC reports, but a recent article [9] whose lead author was the Co-chair of the IPCC Working Group 1 (concerning physical sciences) claims that CO_2_ remains in the atmosphere almost indefinitely. The issue of how long CO_2_ emissions remain in the atmosphere has become even more confusing because global warming supporters have coined a variety of terms related to the residence time of CO_2_ in the atmosphere besides the simple residence time, equal to the average time that molecules remain in the atmosphere.

Segalstad reports [10] as follows (with reference number added):

“[The] IPCC defines lifetime for CO_2_ as the time required for the atmosphere to adjust to a future equilibrium state if emissions change abruptly, and gives a lifetime of 50–200 years in parentheses (Houghton *et al*., 1990) [11]. Their footnote No. 4 to their Table 1.1 explains:

*For each gas in the table, except CO_2_, the “lifetime” is defined here as the ratio of the atmospheric content to the total rate of removal. This time scale also characterizes the rate of adjustment of the atmospheric concentrations if the emission rates are changed abruptly. CO_2_* *is a special case since it has no real sinks, but is merely circulated between various reservoirs (atmosphere, ocean, biota). The “lifetime” of CO_2_* *given in the table is a rough indication of the time it would take for the CO_2_* *concentration to adjust to changes in the emissions…”*

Segalstad [12] argues “the IPCC has constructed an artificial model where they claim that the natural CO_2_ input/output is in static balance, and that all CO_2_ additions from anthropogenic carbon combustion being added to the atmospheric pool will stay there almost indefinitely.” In this regard, the IPCC and its supporters have sometimes tried to support their case by arguing, sometimes with models, that CO_2_ once emitted will stay around for quite some time in one reservoir or another and will continue to pass between reservoirs until it is ultimately transported into ocean sediments. They have sometimes argued that it will take many years for the system to fully return to pre-industrial levels. Although this is probably true, the immediate question from an economic and environmental standpoint is the average residence time of CO_2_ in the atmosphere since that is the only CO_2_ reservoir where it may impact the greenhouse effect. It is possible that CO_2_ emissions might have adverse effects in other reservoirs, of course, but this is highly unlikely in that it represents food for plants and in Segalstad’s view there are extremely large, multiple buffering systems found in the oceanic reservoir [8]. But it is the atmospheric reservoir that is being discussed here. Accordingly, the atmospheric residence time used here is based only on measurements of the air reservoir. The residence time measured by Segalstad [8,10] appears to fit that description. The relevant issue, of course, is what assumptions the models the IPCC and economists actually used were.

There are three different carbon isotopes, carbon 12, 13, and 14, usually denoted as ^12^C, ^13^C, and ^14^C. An important characteristic of plants is that they prefer to use CO_2_ with lighter carbon isotopes, namely, ^12^C. Hence there is a difference in the isotopic composition of CO_2_ derived from plants living or dead compared to other sources (such as volcanic activity), and therefore in the fossil fuels derived from these plants, and in the CO_2_ produced by burning them in one form or another. Specifically, carbon in plants has a lower ^13^C/^12^C ratio. When fossil fuels are burned carbon from the ancient plants from which the fuels were formed is released as CO_2_ into and mixes with the atmosphere, thus lowering the ^13^C/^12^C ratio there. By measuring the isotopic composition of CO_2_ in the atmosphere it is possible to estimate the percentage of CO_2_ derived from plants and to infer the residence time of CO_2_ in the atmosphere. Most but not all anthropogenic CO_2_ emissions come from burning fossil fuels.

The question at hand is whether these measurements correspond to the IPCC assumptions in this regard. If not, there would appear to be something of great importance wrong with their assumptions or hypotheses. The short answer to this question is that the isotopic CO_2_ analysis presented by Segalstad [8,10] are not consistent with the IPCC assumptions in several important regards, and therefore these IPCC assumptions are not scientifically valid assuming that Segalstad’s data and analyses of them are correct.

^13^C/^12^C isotope ratios are usually expressed as δ^13^C values, defined as the standard-normalized difference from the Pee Dee Belemnite (PDB) standard and calculated as follows [13]:

$$\delta^{13}C_{\text{Sample}}=(\frac{{}^{13}C/{}^{12}C_{\text{sample}}}{{}^{13}C/{}^{12}C_{\text{PDB}}}-1)\times 1000$$

This value is expressed in parts per thousand or per mil (“0/00”).

Using an isotopic mass balance analysis, Segalstad [8,10] finds that CO_2_ from burning fossil fuel constitutes only at most 4 percent of atmospheric CO_2_ and that the RT for CO_2_ in the atmosphere is only a little over 5 years. Essenhigh’s research [14] supports some of these findings according to Segalstad [12], who describes his methodology as follows [15]:

“The December 1988 atmospheric CO_2_ composition was computed for its 748 GT C (GT = 10^15^ g) total mass and δ^13^C = −7.807 for 3 components: *(1)* natural fraction remaining from the pre-industrial atmosphere; *(2)* cumulative fraction remaining from all annual fossil-fuel CO_2_ emissions; *(3)* carbon isotope mass-balanced natural fraction. The masses of component *(1)* and *(2)* were computed for different atmospheric lifetimes of CO_2_.”

A more recent reference is [16].

Slide 19 of [8], which Segalstad entitles “Proof from Isotopic Mass Balance,” summarizes his results. His slide relates atmospheric CO_2_ lifetime to atmospheric CO_2_ mass in the atmosphere by the three components listed above by him. In his Slide 18 Segalstad [8] presents data showing that the expected δ^13^C value, if 21 percent of atmospheric CO_2_ comes from burning fossil fuels and CO_2_ has a long lifetime in the atmosphere, would be −11, which he points out is inconsistent with the real world measurements shown in his Slide 19 [8] of −7.807 in December, 1988 as well as in [17]. This is an example of a prediction based on IPCC findings/assumptions that does not correspond to a real world observation, assuming that Segalstad’s data and analysis are correct. Finally, Segalstad’s Slide 20 in [8] shows that the CO_2_ from fossil fuel sources constitutes less than 4 percent of total atmospheric CO_2_, rather than the 21 percent used by the IPCC [8].

**Figure 1.**
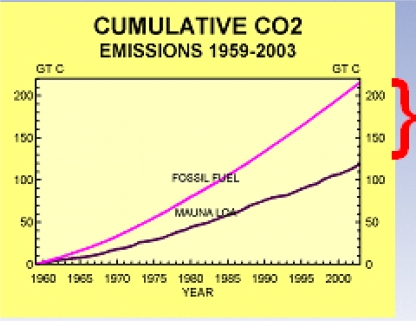
Measured Mauna Loa CO_2_ measurements showing 50% error *versus* the cumulative expected CO_2_ level from burning fossil fuels. Source: [8], Slide 14.

**Figure 2.**
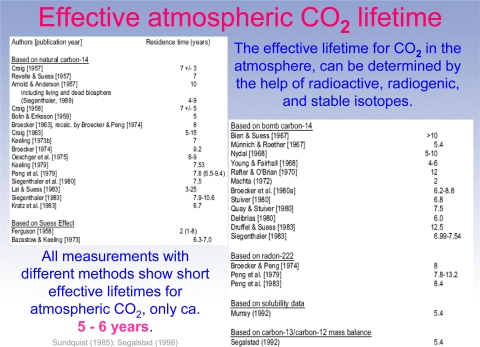
Effective lifetime for CO_2_ in the atmosphere based on a variety of methods. Source: Sundquist [18] and Segalstad [10], as presented in [8], Slide 23.

Segalstad’s findings are bolstered by several other real world observations. One is that climate researchers have long been mystified by the fact that the Mauna Loa CO_2_ measurements can only account for about half of the anthropogenic emissions (as shown in Figure 1), so they have long sought a “missing sink” for CO_2_. Interestingly enough, Segalstad’s analysis shows why this may have occurred, as shown in his slide 21 of [8], where RTs in the range of 50 to 200 years would result in about half the mass of CO_2_ in the atmosphere, as shown by the red circle in his Slide 21, which is remarkably consistent with the “missing sink” that many researchers have looked for in vain. The existence of this problem is another failed prediction of the IPCC hypotheses. The other finding is that dozens of researchers using a variety of techniques have found RTs that are roughly consistent with the 5–6 year time frame, as shown in Figure 2.

The significance of Segalstad’s findings cannot be overestimated assuming his data and analysis are correct. Four percent of atmospheric CO_2_ is in the noise level and contrasts sharply with the IPCC’s 21 percent estimate. The 5+ years RT explains why four percent is reasonable and also contrasts greatly with the IPCC’s apparent assumptions. Segalstad’s findings cast great doubt on some of the most important IPCC assumptions concerning CO_2_ (as noted by Segalstad [8]) as well as on the indirect assumptions made by most if not all economists who have attempted to value the economic benefits of CO_2_ emissions control. Among many other observations, Segalstad points out that a RT of 5 years implies that about 135 GT C is exchanged out of the atmosphere each year. “This is *far more* than the ∼7 GT C annually released from fossil fuel burning” [8]. Further, “anthropogenic CO_2_ is less than ½ W/m^2^, less than 0.1 percent, judged from C isotopes. Clouds are a real thermostat, with far more temperature regulating power than CO_2_” [8]. He does not mention it, but it appears widely agreed that the atmospheric models used by the IPCC do not handle clouds very well.

An important point here is that water can absorb less CO_2_ as temperatures rise. So an alternative explanation to the IPCC hypothesis that the observed rise in atmospheric CO_2_ is due to anthropogenic emissions is that ocean temperatures have at some time in the past warmed enough so that some of its CO_2_ is now being out gassed.

The issue of CO_2_ residence times in the atmosphere is of great importance to economic analyses of the benefits of climate mitigation since it determines the period of time during which the economic benefits of emissions reductions would take place. Short atmospheric residence times would mean that the economic benefits of emissions reductions would occur over a much shorter period than if residence times were very long. The greenhouse effect of added CO_2_ in the natural world is only effective when the CO_2_ is in the atmosphere, not when it is in plants or the oceans, or in ocean sediments. Added CO_2_ (or the carbon in it) in plants or oceans may have important effects, of course, but not for the greenhouse effect on global temperatures. The economic implications of this will be spelled out in more detail in Section 2.6.1 below.

The observed increasing atmospheric CO_2_ levels may be primarily due to increasing ocean temperatures hundreds of years ago since water cannot absorb as much CO_2_ at higher temperatures. In other words, the CAGW supporters may have reversed cause and effect. Instead of increases in CO_2_ causing rising temperatures, rising temperatures result in higher CO_2_ in the atmosphere, probably with a time lag. It is not known exactly what the lag time might be for changes in water temperatures to result in increases or decreases in out gassing from or absorption of CO_2_ by the oceans. Research shows, however, that at the bottom of an ice age 250 thousand years ago CO_2_ increased with about an 800 year lag after temperatures increased [19]. So the current increases in CO_2_ levels might be due to increases in temperatures 800 years ago or could be a result of anthropogenic emissions. Or since much of Earth’s CO_2_ appears to be derived from and vary with changes in volcanic activity [8], this may be another possibility.

In sum, there appears to be substantial evidence that CO_2_ residence times in the atmosphere are about 5 years, that only a small percentage of CO_2_ from fossil fuel sources can be found in the atmosphere, that there are alternative natural explanations for rising CO_2_ levels, and that there is little reason to think that CO_2_ emissions from fossil fuel burning are likely to be a significant cause of the gradually increasing atmospheric CO_2_ levels.

### 2.3. Hypothesis 2: Increases in Atmospheric CO_2_ Levels Interact with the Major Greenhouse Gas, Water Vapor, to Create a Large Positive Feedback Capable of Causing Catastrophic Global Warming

**Figure 3.**
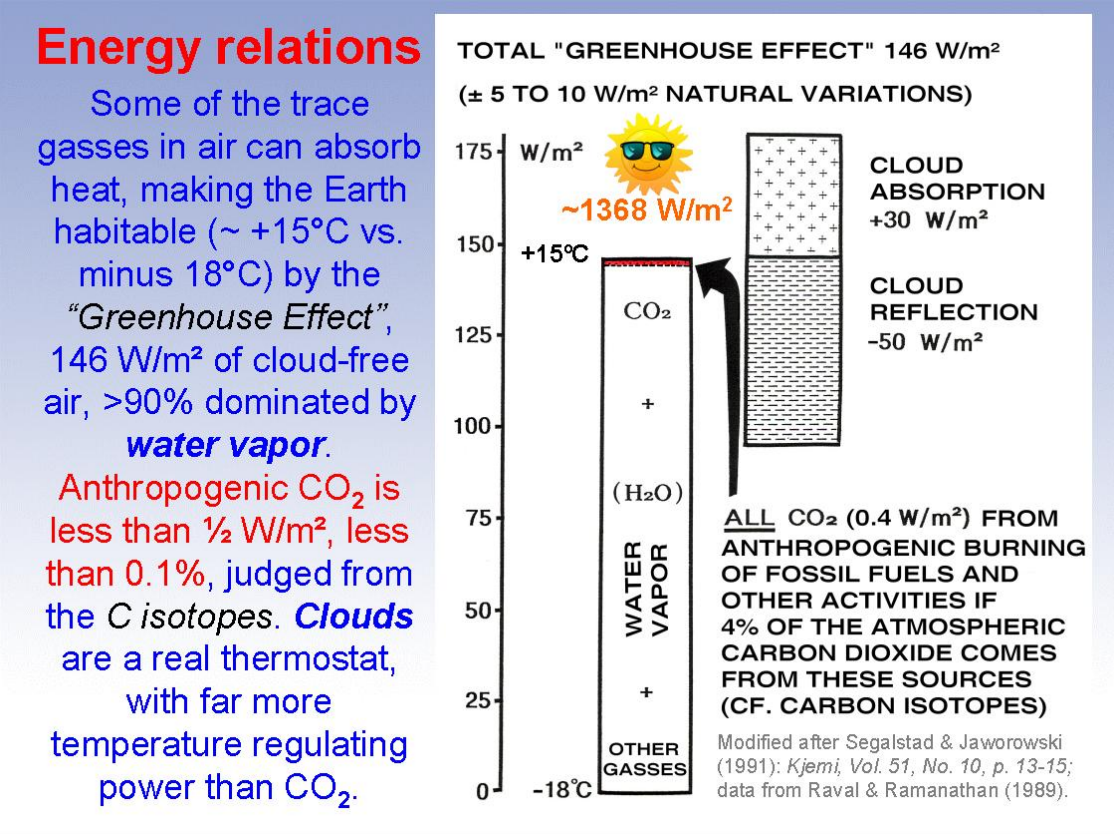
Major role of water vapor and very minor role of CO_2_ in the greenhouse effect. Source: [8], Slide 18.

Undoubtedly the most controversial of the hypotheses underlying the UN’s conclusions is the question of feedback. The IPCC and all the computer models it uses assume that there is a strong positive feedback between an increase in trace greenhouse gases in the atmosphere (such as CO_2_) and the major greenhouse gas (water vapor). Without this strong feedback there is no real basis for CAGW since the atmosphere contains only trace amounts of other greenhouse gases besides water vapor, and the climate sensitivity factor (CSF) for the effect of doubling CO_2_ on global temperatures alone is widely believed to be small (as discussed below and in Section 2.3.1.4). More than 90 percent of the greenhouse effect is due to water vapor and is significantly affected by the extent of cloud cover; CO_2_ from fossil fuel burning does increase the atmospheric CO_2_ level slightly, but represents less than 0.1 percent of the greenhouse effect (as illustrated in Figure 3) according in Segalstad [8].

Archibald [20] points out that “the effect of carbon dioxide on temperature is logarithmic and thus climate sensitivity decreases with increasing concentration,” so that further increases in atmospheric CO_2_ would have very little effect on global temperatures. Further, he finds that:

“The first 20 ppm of carbon dioxide has a greater temperature effect than the next 400 ppm. The rate of annual increase in atmospheric carbon dioxide over the last 30 years has averaged 1.7 ppm. From the current level of 380 ppm, it is projected to rise to 420 ppm by 2030.

“The projected 40 ppm increase reduces emission from the stratosphere to space…This… equates to an increase in atmospheric temperature of 0.04 °C.

“Increasing the carbon dioxide content by a further 200 ppm to 620 ppm, projected by 2150, results in a further 0.16 °C increase in atmospheric temperature.

“Since the beginning of the Industrial Revolution, increased atmospheric carbon dioxide has increased the temperature of the atmosphere by 0.1 °C.”

**Figure 4.**
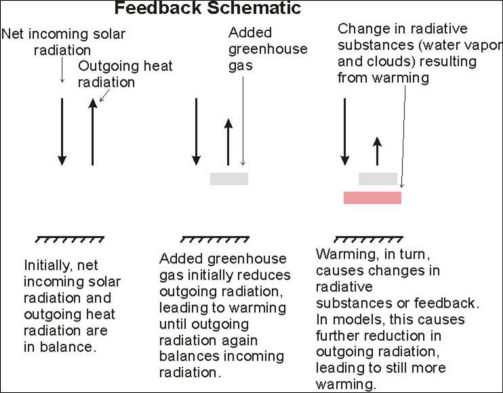
Schematic explanation for possible feedback effects. Source: Lindzen [21].

Figure 4 is a schematic of how this feedback might take place to amplify the effects of increasing atmospheric CO_2_ levels. By assuming a strong feedback, the IPCC AR4 report [5] and the models it uses reach the conclusion that the climate sensitivity factor (CSF) relating the increase in global temperatures (in degrees centigrade) to a doubling in the level of atmospheric CO_2_ is between 2 and 4.5 °C with a best estimate of 3 °C. By using the IPCC or related model results, economists have implicitly used these estimates in determining the benefits of CO_2_ emissions mitigation. Now the major scientific issue is whether this hypothesis that there is strong feedback is supported by the scientific method on the basis of real world data.

#### 2.3.1. Four Critical Comparisons with Real World Data

There are at least four particularly telling physically-based basic comparisons which can be made to determine the scientific validity of the UN strong feedback hypothesis. According to the scientific method an inconsistency even in one of these comparisons means that the hypothesis should be rejected from a scientific viewpoint. In order to determine this it is necessary to compare the physical effects of the assumed strong feedback with actual real world data.

The CAGW concerns are based on a hypothesis that there are significantly positive feedbacks from the effects of the CO_2_ increase on water vapor and clouds that would greatly amplify the greenhouse effect of the increase in CO_2_. This is the key hypothesis which can be compared with real world data to determine whether the IPCC findings are valid or not. Although there are other tests (see [22], Slide 44), hypothesis 2 requires that each of the following four observations discussed in this subsection are present:

1. The atmospheric response times for volcanic sequences would be longer than they would be without the UN’s high positive feedback hypothesis.
2. There is a tropical hot spot in the upper troposphere.
3. There is a heating of the oceans.
4. Observed outgoing radiation fluxes from the earth decrease with increases in sea surface temperatures.

Each of these tests will be discussed in turn, with those depending on the most recent data discussed last.

##### 2.3.1.1. The Atmospheric Response Times for Volcanic Sequences Would Be Longer than They Would Be without the UN’s Strong Positive Feedback Hypothesis

If climate sensitivity is as high as the UN claims, it should show up in the atmosphere’s response time to volcanic eruptions. The reason for this is that climate sensitivity is also a measure of how tightly air and sea temperatures are coupled. High sensitivity is associated with weak coupling, allowing the establishment of significant disequilibration of the sea surface temperature. To quote Lindzen’s Slide 45 [22]:

“Another line of inquiry involved noting that the time for the ocean to respond to a change in forcing increased as climate sensitivity increases. This may seem counter-intuitive, but the idea is simple. Sensitivity is essentially a ratio of a change in temperature to a change in energy flux. High sensitivity means that a change in temperature is accompanied by a small change in flux. However, a small flux takes longer to change the temperature of the ocean. In any event in papers published in 1994 and 1998, we noted that in sensitive climates, a sequence of volcanoes would lead to a secular cooling, but in an insensitive climate, the volcanoes would simply produce transient 1–2 year dips in temperature. The record seems to favor the dips.”

Lindzen provides two references: [23,24]. A 1997 presentation [25] he made at a US National Academy of Sciences symposium clarifies this argument as follows with regard to the volcanic sequence between Krakatoa in 1883 and Katmai in 1912:

“The results show that for sensitive climates (>0.6 °C for a doubling of CO_2_), each volcano builds on the residual base of earlier volcanoes leading to a substantial long-term cooling (∼0.5 °C cooling between 1883 and 1912). For low sensitivity, the response consists in essentially independent ‘blips.’ The observed temperature record certainly shows nothing more than isolated ‘blips.’”

##### 2.3.1.2. There Is a Tropical Hot Spot in the Upper Troposphere

According to Chapter 9 of the IPCC AR4 report [26] all the IPCC climate models predict that there should be a hot spot in the upper troposphere about 5–12 km above the Earth’s surface in the tropics caused by increased evaporation from warmer oceans leading to the accumulation of higher concentrations of water vapor in the upper troposphere, and thereby generating the critical positive feedback reinforcing the small amount of warming that can be caused by increasing CO_2_ alone. The feedback creates the hotspot and is responsible for much of the temperature rises predicted by the IPCC models [27]. If non-water vapor greenhouse gases are significantly warming the Earth, the first signs of it are supposed to appear above the tropics. Since no such hotspot has been found, the models and therefore the UN’s hypothesis concerning feedback are wrong [27]. Various efforts have been made to argue that those looking for the hot spot might have missed it, but have not claimed to have actually found it [28,29]. Sherwood *et al*. tried to find every possible source of uncertainty in the observations in order to argue that agreement with the models was marginally possible. The data are based on well established radiosonde technology and hundreds of tests so it quite unlikely that all the tests were wrong [27], so this hardly constitutes a convincing argument. Statistical counter-arguments to the issues raised by Santer *et al*. [28] can be found in McIntyre [30]. Sherwood *et al*. [29] claimed to have found the hotspot using a combination of temperature and wind data. Using wind data is an example of not using the most relevant observational data since temperature is the issue here. The claimed hotspot, however, is much too faint compared to what the IPCC models predict and there is no rationale for using wind data in place of temperature data [27] when temperature is what is being studied. Lindzen reaches similar conclusions [22] in slide 47.

This hotspot test is perhaps the best known of the tests of the feedback hypothesis, and has been widely written about. One of the most comprehensive and detailed discussions may be Evans [27]. The most easily understood non-technical discussion is probably [31]. The failure of the IPCC or others to find the hotspot that they agree should be present is sufficient by itself to determine that the IPCC’s feedback hypothesis is incorrect, and that the feedback effects of increasing CO_2_ are therefore much smaller than claimed by the IPCC.

##### 2.3.1.3. There Is Heating of the Oceans

The added heat believed by CAGW supporters to be generated by increasing greenhouse gas levels in the atmosphere must be stored somewhere. It has not been showing up in the atmosphere in the last decade, so if the hypothesis is valid it must be going into the oceans. But sea temperature data from the ARGO array for 2003–2008 show no increase, so this has not been the case. An extensive discussion of the evidence can be found in DiPuccio [32]. He concludes that:

“In brief, we know of no mechanism by which vast amounts of “missing” heat can be hidden, transferred, or absorbed within the earth’s system. The only reasonable conclusion—call it a null hypothesis—is that heat is no longer accumulating in the climate system and there is no longer a radiative imbalance caused by anthropogenic forcing. This not only demonstrates that the IPCC models are failing to accurately predict global warming, but also presents a serious challenge to the integrity of the AGW hypothesis.”

Similar questions have been raised and conclusions reached by Roger Pielke Sr. in [33] and preceding references noted there. Lindzen [22] argues on slide 48, referencing Schwartz [34,35], that the rate of change of ocean temperatures shows slightly negative feedback. Hansen and colleagues argue [36] that such warming should be occurring.

So the bottom line is that both sides agree that the oceans should be warming if hypothesis 3 is correct, but current evidence is that they are not. So the CAGW feedback hypothesis fails this rather straightforward and simple test against real world data as well.

##### 2.3.1.4. Observed Outgoing Radiation Fluxes from the Earth Decrease with Increases in Sea Surface Temperatures

All the IPCC models show decreases in radiation fluxes leaving Earth with increases in global temperature, as might be expected under their greenhouse hypothesis. Satellite data, however, show an increase in outgoing radiation, which is inconsistent with the high climate sensitivities to increases in CO_2_ and positive feedback so crucial to the UN’s case.

In August, 2009, Lindzen and Choi published a paper [37] which inferred from satellite data that feedback is not positive at all. Using observations from the ERBE instrument on the ERBS satellite, they analyzed the relationship between tropical sea surface temperatures and top-of-the atmosphere heat radiation. This paper has been criticized by Trenberth *et al*. [38]. Lindzen and Choi have responded with a revised paper [39] using additional data from the CERES instrument on the Terra satellite, but with the same general results. Lindzen and Choi’s revised paper [39] concludes that the satellite data implies a climate sensitivity of 0.7 °C, as compared to a CSF for CO_2_ alone of “about 1 °C” [21] without any feedback. (In 1997 Lindzen testified [40] that the CO_2_ only CSF was 1.2 °C.).

Spencer and Braswell [41], also using observational evidence from satellites, in this case from CERES and the AMSR-E instrument on the newer Aqua satellite, also found negative feedback and an amazingly similar CSF of only 0.6 °C. Spencer’s argument can also be found in [42]. What this means is that there is a feedback, but it is negative, not positive as the IPCC maintains. So instead of an increase in the CSF due to feedback, which results in the IPCC assumed approximately 3 °C CSF, there is a decrease from the CO_2_ only value of 1.0 to 1.2 °C down to 0.6 to 0.7 °C.

According to Spencer and Braswell [41], the models have misinterpreted cause and effect. They believe that it is changes in clouds that cause temperatures changes, not the other way around. Very recently, Dessler [43] claims that he has demonstrated that feedback is positive rather than negative. Spencer [44] has responded that Dessler missed his main point, that when cloud changes cause temperature changes it creates the illusion of a positive cloud feedback, but not the actuality. Although the Spencer/Dessler debate may not be over at this writing, all the other existing tests described above also all argue for a much lower CSF. Although the analyses of the first three do not specify a more appropriate number, Spencer/Braswell [41] and Lindzen/Choi [39] are consistent with them and are thus very plausible.

##### 2.3.1.5. Conclusions from the Four Tests

The conclusions are the same in each of these four cases: The UN feedback hypothesis is not supported or even partially supported by these comparisons with real world data. As Lindzen recently observed [45] with regard to his findings on comparison 4 above, “In a normal field, these results would pretty much wrap things up, but global warming/climate change has developed so much momentum that it has a life of its own—quite removed from science.”

The data are far from perfect, of course, perhaps in part because of a lack of effort to gather it. But the available evidence based on empirical data (rather than models) tell the same story. This means that the hypothesis should be rejected scientifically based on current information. Future testing could lead to other conclusions, of course, but for now rejection is the only rational course of action. It is quite possible that further research may yield a different conclusion, but as of the time that this is being written the evidence strongly supports a negative feedback and a much lower CSF, and this is why a much lower CSF will be used in this paper and why I believe it needs to be by other analysts as well. The important implications of this change in the CSF for the analysis of the economic benefits of CO_2_ emissions reductions will be discussed in Section 2.6.2 below.

It may be useful to provide some explanation why it is that feedback is negative. One explanation is that the IPCC models are not able to represent the effects of clouds very well. There is some evidence, however, that clouds play a very important role and that their net effect on a worldwide basis may be negative [46].

### 2.4. Hyposthesis 3a: Changes in Global Temperatures Are Primarily Influenced by Rising Levels of GHGs Other Than Water Vapor in the Atmosphere

If CO_2_ has such a major role in determining global temperatures as the IPCC and CAGW supporters claim, it should be possible to discern its influence in the available related real world data. This line of inquiry is consistent with the theme of this article that reliance should be placed on real world data rather than computer models in determining the validity of hypotheses concerning climate. The comparisons made in this section do not prove the validity or invalidity of the various hypotheses offered by CAGW supporters. But they do serve as a credibility check on whether the above findings that the two key hypotheses 1 and 2 are invalid are likely to be correct. If there is clear evidence that hypothesis 3a is correct, one might reasonably suspect that the conclusions with regard to hypotheses 1 or 2 might be incorrect despite the overwhelming real-world evidence that the invalidity conclusions are correct, and vice versa.

Five lines of evidence will be presented in this section in the following subsections:

2.4.1 Correlations of various physical attributes with global temperatures.
2.4.2 Correlations of global temperatures with other explanations for variations.
2.4.3 Decrease of temperatures during periods of rising CO_2_.
2.4.4 Increases in satellite-measured temperatures show no indication of CO_2_ influence.
2.4.5 Lack of influence of CO_2_ on temperatures over the last three million years.

#### 2.4.1. Correlations of Physical Attributes with Global Ground-Based Temperatures

A review of the available data correlating CO_2_ levels with global temperatures over the last 110 years makes hypothesis 3 highly suspect. Perhaps the simplest way to demonstrate this is to correlate various factors with global temperatures. One such study [47] found the following for three such factors (see Table 1):

It is very clear that the strongest correlation is between the ocean warming index consisting of the Pacific Decadal Oscillation plus the Atlantic Multidecadal Oscillation (PDO + AMO) and temperatures; the next strongest is with total solar irradiance (TSI), and the weakest is with CO_2_. In fact, CO_2_ has no explanatory power over 1998–2007 decade according to this analysis.

#### 2.4.2. Correlations of Global Temperatures with Other Explanations for Variations

**Figure 5.**
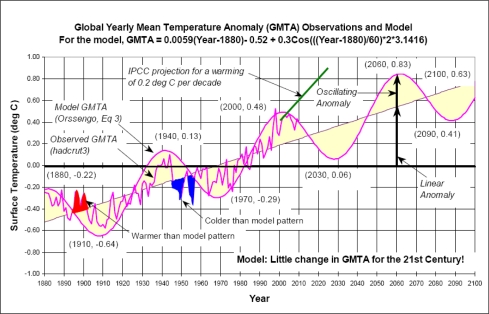
Graph depicting Orssengo’s hypothesis concerning global temperatures. Source: Orssengo [48].

Another hypothesis by Orssengo [48] to explain temperature variation based on ground-based readings over the last 130 years assumes a gradually increasing trend presumably starting from the Dalton Minimum of the Little Ice Age with a 60-year cycle superimposed on it without reference to carbon dioxide or any of the other physical variables tried by d’Aleo [47]. It is illustrated in Figure 5, which compares ground measurements of global temperatures with the hypothesis. This hypothesis may or may not prove to be accurate in forecasting future global temperatures, but Orssengo finds that it has an 88 percent correlation with past temperatures over the period studied. As shown in the figure, there are only a few years in the late 1890s and 1950s that do not appear to fit the hypothesis very well. So no strong support for the IPCC’s CO_2_ hypothesis is evident here either.

#### 2.4.3. Decrease of Temperatures during Periods of Rising CO_2_

A third problem with the CO_2_ hypothesis is that during the period from 1940 to 1970 and after 1998 global ground-measured temperatures fell (see Figure 5) despite rising CO_2_ levels during both periods. There are either other important factors influencing global temperatures besides changes in CO_2_ or the CO_2_ hypothesis is incorrect. Neither explanation is very supportive of hypothesis 3a.

#### 2.4.4. Increases in Satellite-Measured Temperatures Show No Indication of CO_2_ Influence

**Figure 6.**
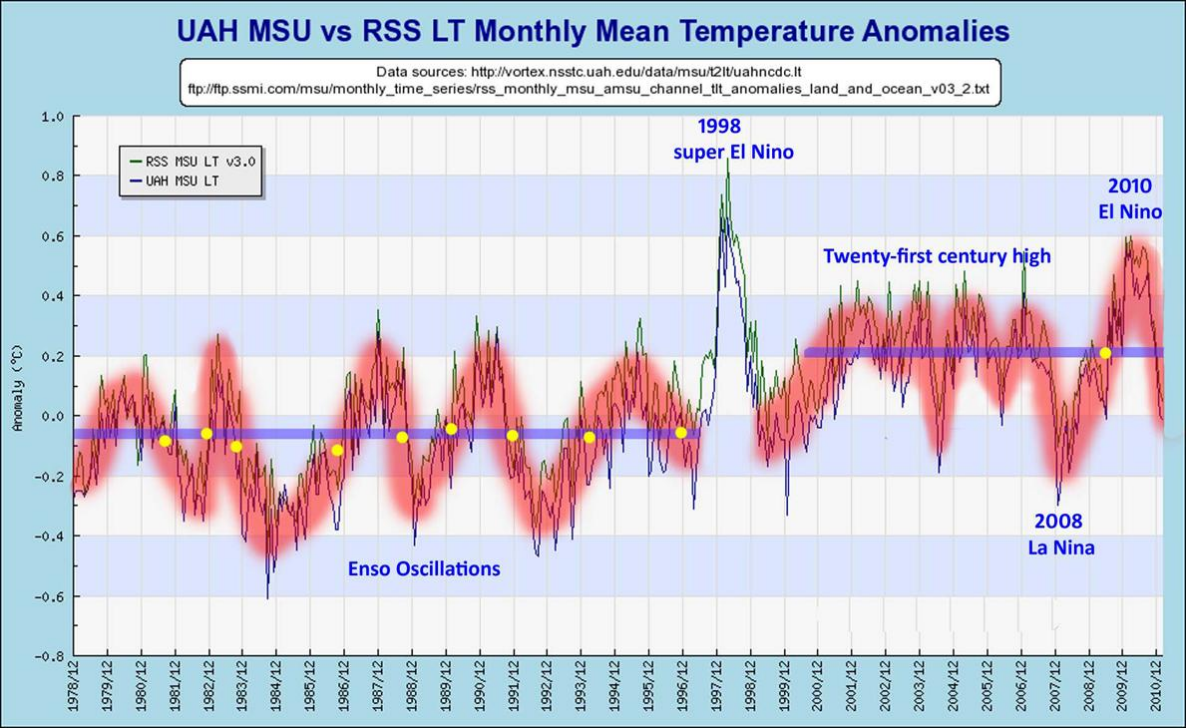
Trend shift in monthly satellite global temperature data. Source: Arrak [50], as updated by him and communicated to the author in March, 2011.

A fourth problem is that even during the periods of rising global temperatures as measured by ground instruments it is hard to see any real correspondence with the gradually rising CO_2_ levels in the satellite temperature data. In fact, there is increasing evidence that much of the increase in ground temperature measurments during the 1980s and early 1990s may be more the result of the urban heat island effect, the poor placement of measuring instruments, and even attempts by the compilers of temperature data to manipulate the ground-based station data than it is of actual temperature increases [49]. The presumed effect of these various cycles is illustrated by a review of satellite data since it started in 1978 to date. Satellite data is not contaminated by the effects of urban heat islands (UHIs), by faulty instrument placement, and by manipulations of the surface data allegedly to “improve” it. The satellite temperature data (which started in 1978) shows a significant increase only in 1998 leaving aside periodic oceanic oscillations (see Figure 6).

**Figure 7.**
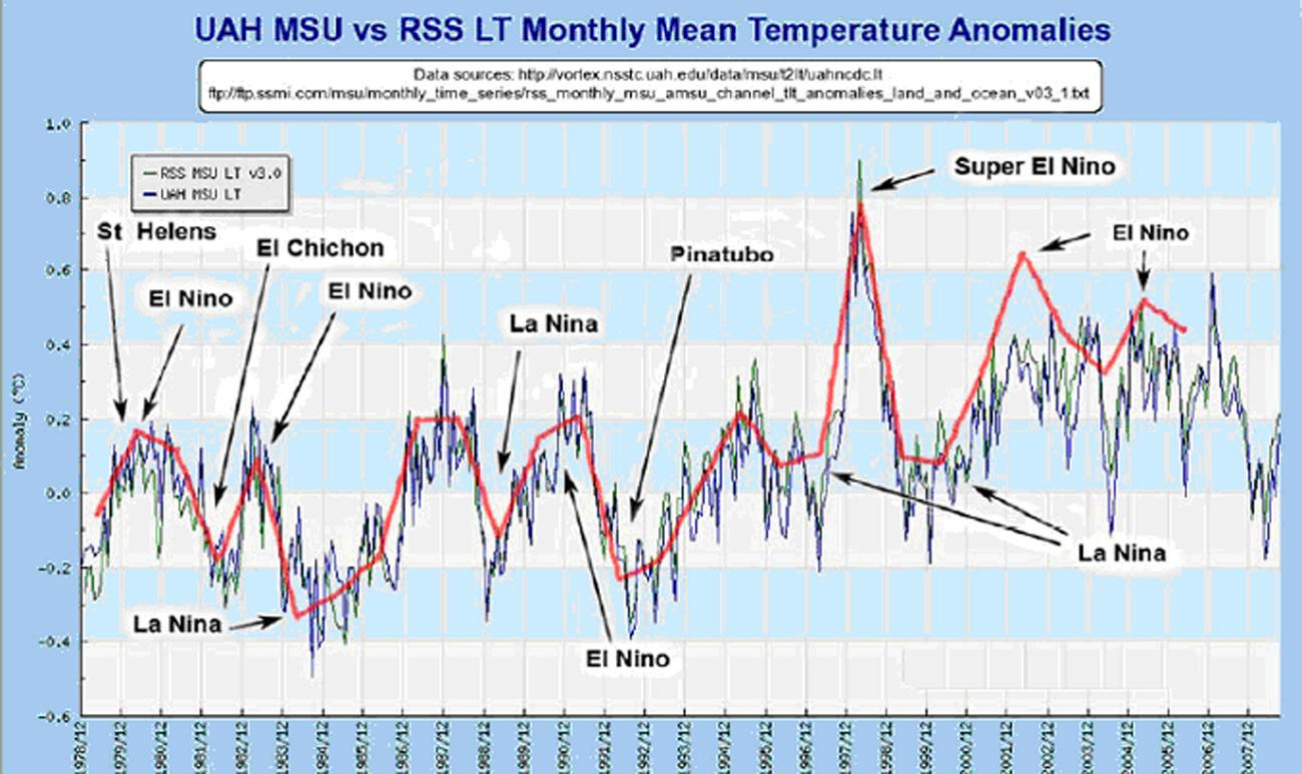
Climate events in relation to satellite global temperatures. Source: Arrak [50].

Surface measurements have so many problems including possible efforts to manipulate them, perhaps to achieve desired temperature trends, and the alleged loss of original data by the Climate Research Unit of the University of East Anglia [51] that it is probably best not to use them for analytical purposes. If CO_2_ increases have had such an important role in increasing global temperatures, it is strange that no such trends are evident in the satellite temperature data. As shown in Figures 6 and 7, most of the evident changes in satellite-measured global temperatures appear to correspond to periodic oceanic oscillations (El Niño and La Niña) in the Pacific, known as ENSO variations, and one major volcanic eruption. The yellow dots in Figure 6 represent the mid-points between each El Nino peak and La Nina valley, and appear to form horizontal lines before and after 1998 in contrast to the continuing fairly steady rise exhibited by atmospheric CO_2_ levels.

In the last 120 years or more there has been a clear variation in global temperatures with roughly alternating warming and cooling periods each lasting about 30 years for a cycle length of about 60 years total. In a 30-year time frame the trends, once started, appear to be form remarkably uniform trends. The reasons for this cycle are not widely agreed on, but any attempt to explain global temperatures needs to explain these observations if it is to be credible. The IPCC climate models do not. One strong possibility is oscillations in sea surface temperatures since changes in the direction of global temperatures seem to have a remarkable coincidence with at least some of these oscillations. Perhaps the most important of these cycles is the Pacific Decadal Oscillation (PDO), although others have been identified in other major oceanic areas (see [52], slides 20–25). The PDO is a long-lived El Niño/La Niña-like pattern that is observed in the sea-surface temperatures (SST) of the Northern and Central Pacific Ocean. Positive (/negative) phases of the PDO are typified by warmer (/cooler) than normal temperatures in the Northeastern and tropical Pacific Ocean and cooler (/warmer) than normal temperatures in the region to the southwest of the Aleutian Islands. It is important to note that while the El Niño/La Niña oscillation varies on a time scale of 4–5 years, the PDO variations are governed by a time scale that is much longer. The immediate point here is that both the PDO and global temperatures have recently turned negative in recent years. Similarly, both turned positive in the 1970s. The reasons for this are speculative at best, but the correlation appears to be overwhelming for the limited period for which we have much data. One possibility is variations in solar output, but much more complicated hypotheses have been proposed [52]. It is worth noting, however, that human concerns about climate change appear to have followed these PDO variations quite closely with concerns about global cooling and a possible new ice age near the end of the last PDO cooling period in the 1970s and concern about global warming in the 1990s and 2000s.

So what would appear to be one of the best “explanations” for the observed changes in global temperatures is provided by the PDO together with ENSO. In fact, major changes in the PDO from positive to negative and back appear to coincide almost exactly with observed changes in global temperature trends over 20–30 year timeframes, as hypothesized in Figure 5.

These graphs are very interesting in another respect. This is that from the beginning of the satellite data in 1978 until 1997 there is no indication that the data varies as a result of changes in CO_2_. Ambient CO_2_ levels were increasing throughout this period yet global satellite-measured temperatures remained in a narrow band with little apparent increase. Further, the spike in temperatures in 1998 appears highly unlikely to have been caused by changes in CO_2_ levels since they vary only very slowly rather than exhibiting the sharp spike seen here. Similarly, the period 1999 to 2006 shows another narrow but higher band of temperatures with no increase during the period. Finally, the period 2007–2009 shows a strong downward trend in temperatures, which is surely not related to steadily increasing CO_2_ levels in the atmosphere. Thus it is very hard to see any effect during the period 1978 to 2009 that can reasonably ascribed to changing CO_2_ or GHG levels. This is in marked contrast with ground level measurements such as the HadCRUT series, which show a marked increase in temperatures through 1998 (see Figure 5) but not thereafter. One possible explanation for this apparent inconsistency between the ground level measurements and satellite data is that ground level measurements may inevitably be compromised by the urban heat island effects, which presumably increased rapidly during the period due to rapid urbanization in many parts of the world.

Given all this, it is hardly surprising that several physical attributes and one model have much higher correlations than does carbon dioxide, which the UN IPCC thinks is the best explanation for increasing global temperatures.

#### 2.4.5. Lack of Influence of CO_2_ on Temperatures over the Last Three Million Years

From a much longer term perspective over the last 3 million years of Earth history, CO_2_ also appears to have played a very limited role in setting interglacial temperatures according to Marsh [53]. Temperatures during past interglacial periods were higher than today most likely as a consequence of lower global cloud albedo due to periodic decreases in cosmic ray flux reaching the Earth’s atmosphere, he believes.

Although the research summarized in this subsection does not disprove hypothesis 3a, it certainly casts considerable doubt on it. The apparent invalidity of hypotheses 1 and 2 suggests why hypothesis 3a may not be more successful.

### 2.5. Implications of Shorter Residence Time and Lower CSF for CAGW

The much shorter residence time for CO_2_ in the atmosphere and the lower CSF determined above have important implications for the IPCC postulated adverse effects of CAGW. The shorter residence time suggests that the fossil fuel contribution to increasing the level of Earth’s atmospheric CO_2_ is actually quite small and thus unlikely to produce CAGW effects other than those experienced over the last three million years. The effects on the atmosphere of such contributions appear to be relatively short-lived, very small compared with the vast CO_2_ absorption capacity of the oceans, and unlikely to result in runaway catastrophic global temperature effects.

The much lower CSF suggests that even the highly doubtful doubling of atmospheric CO_2_ levels would produce only about a 0.6 to 0.7 °C increase in global temperatures. This is less than the increase in global temperatures since the Dalton minimum of the Little Ice Age in the early 1800s. Earth has seen increases of this magnitude during the current interglacial period before and there is no evidence of major CAGW effects as described by CAGW believers.

On the contrary, the evidence is that during interglacial periods over the last 3 million years the risks are on the temperature downside, not the upside. As we approach the point where the Holocene has reached the historical age when a new ice age has repeatedly started in past glacial cycles, this appears likely to be the only CAGW effect that mankind should currently reasonably be concerned about.

Earth is currently in an interglacial period quite similar to others before and after each of the glacial periods that Earth has experienced over the last 3 million years. During these interglacial periods there is currently no known case where global temperatures suddenly and dramatically warmed above interglacial temperatures, such as we are now experiencing, to very much warmer temperatures. There have, of course, been interglacial periods that have experienced slightly higher temperatures, but none that we know of that after 10,000 years experienced a sudden catastrophic further increase in global temperatures. The point here is that there does not appear to be instability towards much warmer temperatures during interglacial periods. There is rather instability towards much colder temperatures, particularly during the later stages of interglacial periods. In fact, Earth has repeatedly entered new ice ages about every 100,000 years during recent cycles, and interglacial periods have lasted about 10,000 years. We are currently very close to the 10,000 year mark for the current interglacial period. So if history is any guide, the main worry should be that of entering a new ice age, with its growing ice sheets, that would probably wipe out civilization in the temperate regions of the Northern Hemisphere—not global warming. The economic damages from a new ice age would indeed be large, and almost certainly catastrophic. Unfortunately, it is very likely to occur sooner or later.

There is no real evidence that this three million year old periodicity in global temperatures has or will change any time soon. And if it did, it would be good rather than bad for humans in that it would mean that there would be less of a threat of a new ice age, which would surely be worse for human economic activity than further minor warming.

### 2.6. General Conclusions Concerning the Economic Benefits of GHG Mitigation

The conclusion from this analysis is that hypotheses 1 and 2 are invalid based on the best current data, and that hypothesis 3a is of doubtful scientific validity and casts still further doubt on the validity of 1 and 2. As mentioned in Section 1.2.2, however, these conclusions are all subject to case-by-case analysis to determine the validity and relevance of the data used. But as a result, the benefit estimates made by many economists for reducing CO_2_ emissions appear to need to be greatly reduced to account for the effects of the reduced CSF and the much shorter residence time of CO_2_ in the atmosphere.

None of the economists and other policy analysts who have attempted to determine the economic benefits of climate change control reviewed here has given any indication that I have been able to find that they checked whether the physical science inputs they directly or indirectly used were valid science based on their correspondence to real world data using the scientific method.

Benefit estimates are based on the economic effects of the temperature increase avoided by decreasing emissions due to mitigation. The emissions decrease from mitigation is assumed to reduce the ambient or atmospheric levels of CO_2_ that would otherwise exist. This ambient CO_2_ reduction is then assumed to result in a temperature decrease using the assumed CSF. But if the emissions reductions from mitigation have little effect on ambient CO_2_ levels (as would be the case since hypothesis 1 appears to be invalid), then there would be little or no change in ambient CO_2_ levels and hence little change in temperatures, especially at the reduced CSF level.

#### 2.6.1. Effect of Reduced CO_2_ Residence Time on the Economic Benefits of Emissions Control

It appears that the effect of the economic correction resulting from the apparent invalidity of hypothesis 1 is very large, as discussed in Section 2.2 above. According to Segalstad’s analysis [8,10,12] summarized there, CO_2_ emissions remain in the atmosphere for a little more than 5 years, on average, after which they are absorbed by the oceans, plants, or ocean sediments, where they would have no effect on global temperatures through greenhouse gas effects. The economic benefits from mitigation efforts carried out in any one year would accordingly only be during the following five years on average.

Lacking clear information on what assumptions were actually used concerning residence time by the IPCC and the models they used, or those used directly or indirectly by previous economic analyses, I propose to use a correction factor equal to Segalstad’s 5 year residence time, as developed in Section 2.2, divided by 125 years (taking the 1990 IPCC report’s midpoint between 50 and 2000 years), or 0.04. If what appears to be Solomon *et al*.’s more than 1000 year estimate (see [9] and the discussion of it in Section 2.2) was used in the AR4 models, the correction factor would be less than 0.005, so the correction factor used may need to be almost a further order of magnitude less.

The argument is that an added ton of CO_2_ prevented from entering the atmosphere will mean that atmospheric CO_2_ will be decreased for about five years relative to what it would otherwise have been during that period. This decrease will decrease global temperatures very slightly compared to what they would otherwise have been based on the CSF during that period, but on average not thereafter. But at the end of the period, the decreased atmospheric CO_2_ levels will have reverted on average to what they would have been without the emission reduction.

A good argument can be made that because the economic benefits of control should be discounted that the above fraction overstates the correction that needs to be made. Obviously a more refined estimate could be made taking into account the appropriate discounting of future reductions of economic benefits that would no longer occur, but it would be messy given the different discount rates that previous economic analysts have used, the sketchy basis for what the IPCC models and each previous economic analysis assumed in the regard, and the necessity to compute individual corrections for individual analyses. In the case of those economic analyses that have used near-zero discount rates, however, the discount rate adjustment would also be near zero. And these are probably the only analyses for which there is likely to be much question as to whether the remaining economic benefits after hypotheses 1 and 2 corrections have been made might still be significant. Also the overstatement of the residence time correction due to ignoring the discount rate correction may be counterbalanced by the assumption that the IPCC computer models used in earlier economic analyses effectively only assumed a 125 year residence time when in reality they may have assumed that it was over 1000 years.

#### 2.6.2. Effect of Reduced CSF on the Economic Benefits of Emissions Control

A lower CSF means a lower temperature increase, which also means lower economic benefits of control. Further research may show that the CSF is a little higher or a little lower, but it is clear that the UN IPCC CSF estimates are much too high based on the four real world tests summarized in Section 2.3.

If valid physical science relationships were used in the case of hypothesis 2, the economic benefits would have been much less, probably less than one-fourth those used on the basis of the CSFs based on the 2007 IPCC best estimate. If the Lindzen/Choi [21,39] and Spencer/Braswell [41] CSF findings are correct, this means that benefit estimates that were based on the IPCC AR4 [5] best estimate of 3.0 °C need to be multiplied by a fraction no more than that determined by either the latest Lindzen [21,39] CSF estimate of 0.7 or the Spencer/Braswell [41] estimate of 0.6, each divided by 3. This yields a correction factor of between 0.20 and 0.23. For simplicity this article will assume this fraction to be less than one-quarter or 0.25. This assumes that the economic benefits can be scaled linearly with temperature. In reality, it appears likely that the benefits rise more rapidly with temperature, particularly at extremely high temperature changes, so one-quarter is an underestimation of the change in the actual revised benefits. One footnote is that a number of the economic benefit studies were done prior to the IPCC AR4, and the earlier IPCC reports used slightly different values for the best estimate for CSF, although the changes were small.

Such reduced increases in global average temperatures do not appear to be large enough for there to be any significant risk of CAGW since even a doubling of CO_2_ levels would result in less than a 0.7 °C increase in global temperatures, which is well within temperature variations during the Holocene and during which no CAGW effects are known to have occurred. Hence by carefully examining the science, it is reasonable to conclude that CAGW effects are highly unlikely and probably impossible. That leaves the non-catastrophic economic benefits from avoiding relatively minor increases in global temperatures. Although extreme increases in global temperatures might have substantial adverse effects, it is not clear in the case of small increases suggested by CSFs of 0.6 to 0.7 °C whether increases of that magnitude would be beneficial rather than harmful. Tol [54] believes they would be beneficial. If significant warming occurred, Northern areas of the Northern Hemisphere would presumably be opened to cultivation and use, as Southern Greenland was in the Medieval Warm Period. The scientific validity of physical science hypotheses such as the value of the CSF can only be determined by comparisons with real world data using the scientific method and not by the use of peer review or atmospheric models or statements by experts. Although Lindzen/Choi [21,39] and Spencer/Braswell [41] have derived their estimates of the CSF by using real world data, that does not prove that their estimates will ultimately prove accurate. They do, however, appear to be much more solidly grounded in real world observations than the IPCC AR4 primarily model-based estimates. And they are consistent with the four negative tests of the high CSFs used by the IPCC AR4 discussed in Section 2.3.

#### 2.6.3. Combined Effect of the CSF and Residence Time Corrections

The net result of combining both the hypotheses 1 and 2 correction adjustments suggest above would be a combined factor of roughly 0.04 times 0.25, or 0.01. This would mean that all benefit estimates that have not adjusted for the postulated invalidities of hypotheses 1 and 2 would need to be divided by about 100. The result would be that most benefit estimates would be so small as not to be worth further discussion.

## 3. The Economic Costs of Climate Change Mitigation

I believe that there are also significant differences in the costs of reducing CAGW than usually assumed by many economists who have examined this question. First, the much smaller CSF and shorter residence times for CO_2_ in the atmosphere explained in Sections 2.2, 2.3, and 2.6 above greatly increase the cost of reducing CO_2_ emissions *per degree* of assumed global cooling achieved since it takes much more of a change in CO_2_ emissions to reduce temperatures by a given quantity. In other words, just as an increase in atmospheric GHG levels increases temperatures much less than assumed by the IPCC, reductions in CO_2_ emissions would be much less effective in decreasing temperatures. Note that this does not change the total cost of mitigation because there will presumably be proportionately less of a temperature increase to mitigate. The second difference is that most efforts to estimate costs of CO_2_ emissions reductions appear to have greatly underestimated the actual costs. The third difference is that with one exception only one of the analysts discussed in this paper looked at alternative less expensive methods for achieving such mitigation should it ever be needed and desired. These three differences will be discussed in the next three subsections.

### 3.1. Why the Effectiveness of Proposed Reductions in CO_2_ Emissions in Reducing Temperature Increases Will Be Much Lower than Assumed by Many Economists

The much lower CSF found by Lindzen/Choi [21,39] and Spencer/Braswell [41] affects the costs as well as the benefits of global warming mitigation. As outlined in Section 2.6, the hypothesized increase in global temperatures would be more than four times less than shown by the IPCC AR4 models because the CSF appears to be at least four times less. If the economic damages are proportional to the temperature increase, then damages (benefits of mitigation) would be more than four times lower. Similarly, the cost of *each degree* of mitigation would be at least four times higher since the effect of lowering CO_2_ levels would be less than one-fourth as much. So the economic benefits of mitigation would be only one-fourth as much while the overall cost of mitigation would remain the same but achieve less than one-fourth the global temperature reduction previously assumed. As a result the benefit-cost ratio of “conventional” reductions in CO_2_ emissions would be less than one-fourth the levels previously calculated, all other things being equal. Thus the temperature increases to be mitigated would be less than one-quarter those assumed in the benefit studies, so the benefits of most proposed mitigation efforts would be less than one-quarter, but the cost of mitigating these damages would remain the same.

Similarly, the much lower and empirically derived residence times for CO_2_ in the atmosphere greatly reduce the effectiveness of CO_2_ emissions reductions in decreasing atmospheric CO_2_ levels and therefore temperatures. There is much less of an increase in temperatures to offset, but the cost of offsetting them would be much the same as has previously been estimated by other analysts.

### 3.2. The Cost of Reducing Carbon Emissions Are Much Higher than Usually Assumed

I have shown elsewhere that using the UN IPCC assumptions the 2 °C EU target is not likely to be achievable even at very high cost [55] (particularly pp. 712–716 and Appendix 1). The acronym ERD means exclusive regulatory decarbonization and is discussed further in Section 3.3 below. The following excerpt summarizes this finding [55] with a reference added:

“Even if climate sensitivity to increased CO_2_ is what the IPCC says it is, the modeling work by Rive *et al*. suggests that it would not only be risky but also very expensive to actually achieve the two degrees Celsius limit using ERD [56]. They find that to obtain a mere fifty percent chance of preventing more than a two degrees Celsius increase would require a global cut of eighty percent from current industrial emission levels by 2050 at a marginal cost of $3,500 per ton of carbon equivalent assuming average projections and “early action” to reduce GHGs. $3,500 is roughly an order of magnitude higher than most previous estimates of marginal costs, presumably reflecting the extremely high cost of rapidly replacing most of the energy producing and using capital stock. An eighty percent cut would imply a reduction per person of about eighty-seven percent below current levels because of predicted world population growth. This appears to be of very doubtful practicality, particularly at the extremely high marginal costs estimated by Rive *et al*., and has a mere fifty percent chance of “success” even in the “ideal” world of modeling. This suggests that in the real world a serious effort to achieve such cuts would be extremely expensive, require worldwide cooperation and an early start, and be much more likely to lead to catastrophe than success….Rive *et al*. furthermore find that if we wait an additional ten years to implement serious emissions reductions, a fifty percent change would not be achievable at all, again assuming “average” projections….The apparent implication is that even under a two degrees Celsius limit and three degrees Celsius sensitivity ERD is a very long shot with little real hope of meeting the two degrees Celsius limit even before taking into account the wide gap that is almost certain to exist between what is actually achieved and what countries and their citizens may agree to do.”

Galiana and Green [57] also argue that most prior cost of mitigation analyses are grossly optimistic. Their arguments are summarized in the following excerpt:

“A ‘thought experiment’ helps to illustrate. Suppose the emission reduction target is an 80% reduction in global emission from current levels by 2100. To reach the 2100 target requires a 1.8% average annual rate of decline in carbon emissions. Now suppose the expected ‘trend’ rate of growth in global world output (GWP) from 2010–2050 is 2.2%. To avoid a reduction in the growth rate of GWP would require a 4.0% average annual rate of decline in the carbon intensity of output (RCIO)….

“If a policy of reducing emissions by “brute force” is adopted, irrespective of technical feasibility, even an increase in the average annual RCIO to 3.6% from its ‘historic’ rate of 1.3% (a very unlikely event in the absence of a technology-led policy) implies a reduction in the growth rate of GWP from the 2.2% ‘trend’ rate to 1.8% for the period 2010–2100. Such a reduction would cost (an *undiscounted*) $86 trillion in 2100 alone and an *undiscounted* $2280 trillion *cumulative* over the 90 year interval. (It is assumed GWP in 2010 is $41 trillion, measured in MER terms.) And even these huge reductions in GWP would not do the trick (meet the emission target) if we cannot push the rate of *decline* in C/GWP up to 3.6% (which is almost triple the “historic” rate).

“The ‘thought experiment’ casts serious doubt on the credibility of estimates of the cost of stabilizing climate. Estimates in the 1 to 3% of global GDP range—or lower (Stern, 2007; IPCC, 2007) are not credible unless there is a *prior focus* on reducing the technology gap. The low-cost estimates reflect a variety of self-serving assumptions. Some models employ an emission scenario baseline that builds in large, automatic improvements in energy technology. Other models include a *carbon-free backstop technology* (often generic) that assures that once the carbon price reaches a specified level there is an unlimited supply of carbon emission-free energy forthcoming. Still others have very high implicit rates of energy intensity decline, ones that would almost surely be physically impossible to achieve. Finally, some models make very optimistic assumptions (ones generally inconsistent with the evidence) about the availability and readiness of carbon-neutral technologies and/or the responsiveness of successful innovation of new energy technologies to carbon prices.

“None of these modeling conveniences or assumptions contribute to a *reliable* approach to estimating the cost of mitigation. Perhaps the most deceptive are models that build-in a backstop carbon-free energy technology, because this effectively assumes away what *is* the problem. Unless a specific effort is made to research and develop, test, and make ready-for deployment scalable carbon emission-free technologies, the cost of mitigation is likely to be as much as an order of magnitude, or more, higher than has been reported.”

Galiana and Green’s order of magnitude cost increase appears reasonable; I am much less optimistic, however, that a research and development effort such as they propose is likely to solve the problem of astronomical costs. The costs of CO_2_ emissions reductions on the scale proposed by CAGW supporters are very large and much higher than usually quoted. If “conventional” mitigation is to be attempted (and I do not believe that it is economically justified or currently needed), some way would need to be found to reduce the costs substantially. Galiana and Green [57] as well as Lomborg [58], based on their work, suggest that the way to do this is to impose a low level carbon tax to generate revenue for technological research to bring down the costs of reducing CO_2_ emissions. Their arguments have some merit, but there is considerable doubt whether throwing even more research money at the climate change problem (even before Obama, the US Government was spending about $5 billion per year on climate-related research [59] will produce the technological progress needed to substantially reduce the costs? Or would more money simply build an even larger and better funded research community to lobby for even more funds? The answer is that research is inherently unpredictable and there is no assurance that even greater amounts will “solve” broadly focused problems such as cancer, or in this case, energy efficiency.

Research and development designed to exploit a promising new technological opportunity is a very different thing than attempting technological improvements over a broad but well researched set of possibilities, none of which offer immediate reason to believe a breakthrough is imminent. The US Government had great success with a narrowly focused Manhattan Project during World War II but in that case there was a narrowly focused goal which attempted to exploit a new technological approach, Such is not the case here, where any number of technologies would need to be explored with none holding out great current promise of major cost reductions.

As an example, it now appears that an important advance has been made by combining horizontal drilling and hydraulic fracturing technology to obtain both oil and natural gas from shale formations. This may promise the US and a number of other countries with suitable such formations the possibility of energy independence from the Middle East, but this breakthrough was not achieved by a large government-run research effort but rather by private initiative over many years. Technological progress requires not just more government research expenditures, but also luck and a willingness to follow valid scientific principles that are needed to carry out good science. The much lower energy costs promised by this particular technological breakthrough also raises the bar for developing new reduced CO_2_ emitting technologies that much higher since the increased production of natural gas and oil using these technologies promise lower fuel costs which any lower carbon emitting alternative would have to meet to be competitive and therefore viable without government subsidies—presumably the goal of Lomborg [58] and Galiana and Green’s [57] proposed research.

### 3.3. Geoengineering as an Alternative to Reducing GHG Emissions Needs to Be Considered

Fortunately, as Lomborg [58] has recognized, there is an alternative to what appears to be a rather futile effort to reduce carbon emissions [55]. This alternative is geoengineering. This promises global temperature decreases (or increases if needed) at a tiny fraction of the cost of carbon emissions reductions with much greater certainty [55,60]. One of the major assumptions made by those advocating climate change mitigation has been that the way to do it is by reducing human-caused emissions of GHGs. Perhaps because CAGW supporters allege that it is increasing GHG levels, particularly CO_2_, that is causing and will cause the increase in temperatures, this assumption has apparently been plausible enough to many people that little effort has been made by most environmental economists (or physical scientists, for that matter) to determine whether such reductions actually would reduce global warming or whether there might be other less expensive or more effective alternatives that would accomplish such a reduction in global warming.

Speaking broadly, most environmental economists who have examined the costs of GHG mitigation have directly or indirectly assumed that (1) reducing GHG emissions will significantly reduce global warming, (2) such reductions are either the best alternative available for this purpose or at least the only one worth examining, and (3) geo-engineering approaches either are not worth examining or can be ruled out for various reasons, particularly “tampering with nature” and the possibility of unintended consequences [55,60]. The major exception is Lomborg [58] and associates [61], who have examined some geoengineering approaches in great detail, and whose views will be discussed further in Section 5.2 below.

The most prominent lower cost alternatives involve geoengineering, which in this case means deliberate modification of the atmosphere by humans. There are a number of varieties involving different approaches towards reducing global temperatures. One of these approaches, involving placing various types of particles into the stratosphere to increase or decrease the reflection of incoming solar radiation [60] so that they will not be washed out by rain, is sometimes called solar radiation management or SRM. Another involves placing salt molecules into air above oceans using ships [61].

Previous research [55,60] resulted in comparisons between SRM and reductions in GHG emissions (see Table 2), referred to as exclusive regulatory decarbonization (ERD). ERD is defined as a strategy for decreasing emissions of greenhouse gases as the exclusive approach used to control global warming. Most current climate change control proposals involve some form of ERD such as emission regulations, emission taxes, fuel economy, bio-fuel standards, and “cap and trade” proposals. ERD assumes that if we could just reduce carbon emissions that humans are putting into the environment, the global warming problem would be solved. I have termed such attempts ERD because most, but not all, involve decreasing various forms of carbon emissions.

As can be seen in the table under marginal costs, rough estimates place the equivalent cost of reducing carbon emissions as 4 to 5 orders of magnitude (that is, 10,000 to 100,000 times) less than the cost by reducing carbon emissions. As discussed in Section 3.2 the actual costs of ERD are probably another order of magnitude higher than shown. These estimates are necessarily rough given that no actual geoengineering experimentation has been done by humans. And various other forms of geoengineering probably have somewhat higher or lower costs. The differences, however, are so large that it is reasonable to assume that at least SRM and possibly some other geoengineering approaches are much less expensive. And most important, the global temperature effects of SRM have been repeatedly demonstrated by nature as a result of major volcanic eruptions in the tropics. Such is not the case for emissions reductions, which have not been demonstrated to actually achieve any temperature reductions. A very important consideration is that with adequate preparation, no actual use of SRM would be needed until there were clear and immediate risks of either decreases or increases in global temperatures that posed a serious risk to humans or the environment. Errors could be quickly corrected by altering the type, quantity, or placement of particles in the stratosphere. Emissions reductions, on the other hand, require a guess decades in advance as to when and how adverse an effect may occur and how much of a decrease/increase in GHGs might be required to mitigate these effects. If the assumed CSF or CO_2_ residence time in the atmosphere prove to be incorrect or people or governments do not fulfill their obligations, it would take several additional decades to correct any errors made because of the long time lag required to alter behavior and significantly change atmospheric GHG levels.

If 80% emissions reductions are to be achieved by 2050, as the Waxman-Markey bill in the US House of Representatives [7] proposed, taking account of population growth and increases in energy use since 1990, the reductions “needed” per person would be almost 90% (see [55], p. 721). Given the rapid spread of new energy using technology such as computers, server farms, cell phones, and other electronic gadgets, this appears more than unlikely.

In reality, most experience to date has been that in political jurisdictions where the most serious energy efficiency efforts have been made, the “best” that has been achieved is that GHG emissions have been held steady because the emissions reductions have been balanced out by increases brought about by demand for increased use by increasing urban populations (see [55], pp. 721–725). Finally, as summarized in Table 2, stratospheric SRM would much more reliably achieve cooling at a small fraction of the huge costs of reducing GHG emissions.

So although SRM and other geoengineering approaches to controlling global warming have obvious problems such as who is to make the decisions and on what basis [62], it appears that they would accomplish the goal of reducing (or increasing for that matter) global temperatures at a far more reasonable cost and to do so fairly promptly and reliably. It would appear desirable for economists to at least analyze this possibility in discussions of the costs of CAGW control.

One benefit of SRM is that it can be used entirely as a very low cost insurance policy until and unless there is strong evidence that global temperatures are increasing (or decreasing) rapidly. This insurance policy should entirely eliminate the need to be concerned about low probability, high consequence CAGW effects since action could be taken if and when needed rather than decades in advance as in the case of ERD.

### 3.4. Implications for the Costs of Climate Change

If economists had carefully considered the scientific validity of the physical aspects of the environmental benefits and the costs of alternatives to emissions reductions they would have probably been less likely to recommend reducing global GHG emissions and more likely to have recommended geoengineering that although controversial has a high likelihood and low cost of controlling global temperatures should that be needed and desired. The possibility of using geoengineering eliminates the argument for pursuing very high cost GHG-mitigation alternatives as a safety hedge against small risks of CAGW outcomes since these low-cost approaches can be implemented very rapidly if they should actually ever be needed and if minimal research has been carried out in advance and a decision-making process has been put in place. Because mitigation measures do not have to be undertaken decades in advance, even these much lower operating costs can be postponed until there should be an evident need.

## 4. Implications for the Benefit-Cost Analysis of Climate Change Mitigation

The broad results of this analysis to this point are that the economic benefits of climate change mitigation appear to be perhaps 1/100th of those assumed by most economic analyses because of real world data showing a much lower CSF and shorter CO_2_ residence times in the atmosphere than assumed by the IPCC. The costs, on the other hand, would appear to be about an order of magnitude higher because of technological and implementation problems recently identified. This means that “conventional” benefit-cost analysis would conclude that the proposed climate change mitigation would be much more likely to fail an economic efficiency test than previously estimated. But the costs of control can be greatly reduced by adopting an entirely different approach to mitigation (geoengineering such as SRM) based on very promising but largely ignored technology. The costs of this promising but so far not carefully validated approach appear to be many orders of magnitude less expensive, albeit with possible new and also little researched risks. These costs are so low that climate change mitigation might actually be economically efficient if global temperatures should start decreasing rapidly (since SRM can be used on the temperature downside as well as the upside). This approach also removes concern about low probability, high consequence events arising from increasing global temperatures since geoengineering options such as SRM can be implemented extremely rapidly with adequate preparations, unlike (probably futile) attempts to reduce atmospheric CO_2_ levels decades in advance, as currently proposed by the UN and some Western governments.

On the other hand, the risk of CAGW appears to be so low (given the low CSF and the short residence time of the fossil fuel contribution to increasing the level of Earth’s atmospheric CO_2_) that it does not appear to be worth even trying to do anything to mitigate it currently, particularly since the world has any number of other problems that appear to be more urgent. But if an economically efficient control effort should ever be needed, which eventually it probably will as we approach the end of the Holocene with a high likelihood of a new ice age, the most economically efficient approach appears to be by using geoengineering such as SRM assuming adequate research and validation has been undertaken beforehand and reasonable implementation approaches are devised. So it would appear worthwhile to undertake these comparatively inexpensive preparatory efforts anyway.

## 5. Some Comparisons with Other Economic Analyses of Climate Change Control

There have been at least 16 economic analyses of climate change control in the literature broadly construed. Table 3 summarizes the economic effects of the increases in global temperatures shown in most of these analyses, based primarily on Tol [54]. In addition, there have been two major government reports: one in Great Britain, Stern [63], and one in Australia, Garnaut [64]. Finally there is a more popular article by Krugman (2010) [65]. Tol [54] summarizes the findings of most of the academic articles/books with estimates of the uncertainty given in brackets, either as standard deviations or as 95% confidence intervals where available. Fragmentary data from the other three studies above are listed at the bottom of Table 3.

Most of the first 13 studies listed in the table find that temperature increases of 1 to 3 °C will result in decreases in GDP (or more accurately GWP) of a few percent. The principal outliers would appear to be those by Tol [54,74], Stern [63], Garnaut [64], and Krugman [65]. Tol [54] believes that minor warming would result in higher growth and is thus one of the principal outliers on the optimistic side. The last three, on the other hand, appear to be outliers on the pessimistic side. Stern has been criticized by Nordhaus [79] for using unreasonably low interest rates to justify what Krugman [65] calls the “big bang” approach, which also applies to the other major government report by Garnaut [64], which uses an even lower rate. I can only agree. The exact effect of the reduction in the value of the CSF on the outcome of each analysis would require detailed reanalysis in each case, but except for Krugman and the two government reports it seems likely that it would make the difference between positive and negative net benefits in most cases. This is the case for the one positive scenario reported by Tol [54].

The next two subsections will summarize two of the most recent analyses by Krugman [65] and Lomborg [58], both of which have unusual and significant features with regard to this analysis and illustrate the profound effect of the basic assumptions by each analyst on their recommendations.

### 5.1. Krugman

Paul Krugman is arguably one of the more influential American economists given his long-running column in the *New York Times*. In 2010 he wrote an extensive summary of global climate economics for the *New York Times Magazine* [65]. Although not in academic format it is probably one of the most widely read articles on the subject to date. So it is worth examining it in some detail for the insight it may provide on economists’ use of physical science information and the influence this has on the conclusions reached.

Krugman’s analysis [65] has a brief three-paragraph summary of his understanding of the physical science aspects. This is unusual and worthy of note since many other economists have either not tried to understand the physical science aspects or at least not reported having tried to do so. So he deserves credit for recognizing the importance of these aspects. He did not provide (reasonably, given the magazine format), however, citations for his viewpoints, so it is unclear where he obtained his views. These views, however, do not ask the crucial questions concerning the consistency of the science with the scientific method emphasized in this article, but are nevertheless important to understanding his argument so are worthy of detailed analysis. His basic points [65] were as follows:

1. There has been an upward trend in global temperatures since the 1970s,
2. “Climate models predicted this well in advance, even getting the magnitude of the temperature rise roughly right.” This “gives them enormous credibility.”
3. “Models based on this research indicate that if we continue adding greenhouse gases to the atmosphere as we have, we will eventually face drastic changes in the climate.”

**Figure 8.**
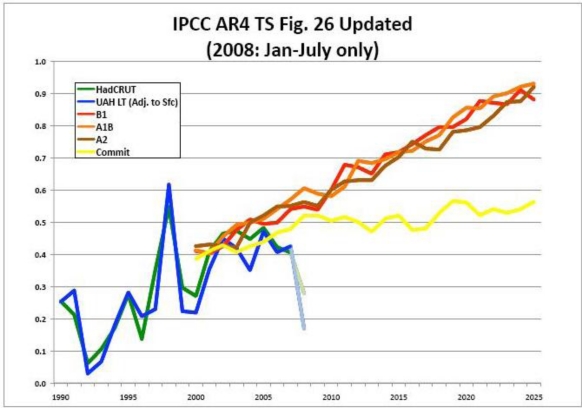
HadCRUT3 and UAH *versus* IPCC model global surface warming. Source: [80], based on a graph prepared by Dr. John Christy.

My comments on these points are as follows:

1. Global temperatures have been trending upwards not just since the 1970s, but since the end of the Little Ice Age (see Figure 5 and Section 2.4.2), with what appears to be a superimposed 60-year cycle. So this upward trend existed long before there were significant anthropogenic GHG emissions, and the increases since the 1970s are hardly the most relevant observational data.
2. It is highly questionable whether the climate models used by the IPCC made any such predictions (see Figure 8, which shows the divergence between various IPCC scenarios shown in red, orange, and brown, with the actual satellite temperature measurements adjusted to surface in blue and adjusted ground temperature data in green; see also the green line in Figure 5). And there is no evidence that the climate models have even managed to correctly hindcast temperatures [4]. But even if they did so forecast, who predicted what and when provides no valid scientific evidence about whether the models can predict the future. So again this is not the most relevant observational data.
3. It is probably true that the IPCC models will continue to produce such results. Whether they have any predictive capability, however, is highly dubious for the reasons discussed in Section 1.2.1 Krugman’s views on the coming apocalypse, however, are of considerable importance because they appear to form the basis on which he bases his final conclusions in the article.

So Krugman recognized the importance of the science, but appears not to have asked the crucial questions necessary to validate his views in terms of whether they reflected the most relevant observational data and the scientific method.

In the remainder of his article, Krugman explains that economists have supported two different approaches to climate change mitigation. He characterizes the first of these as a “policy ramp” approach because it proposes that GHG emissions controls should start slowly and eventually be ramped-up to much higher levels that the proposers believe will provide adequate control to prevent CAGW. (He does not explain why this approach might make sense, but it postpones major expenditures so that the present discounted costs are much less.) He characterizes the second approach as a “big-bang” approach in that emissions controls would initially be much nearer the levels that are thought to reduce emissions sufficiently to allegedly avoid CAGW. He then supports Weitzman’s views (not referenced, but presumably [81]) concerning the difficulty that economics may have in analyzing programs that may have a low risk of uncertain but overwhelming impacts, and uses these concerns plus his own views concerning the importance and magnitude of these alleged CAGW effects of increasing atmospheric GHG levels to support the “big bang” approach.

In other words, Krugman argues that because he has heard that there could be CAGW effects, it is worth disregarding the “standard” ramp-up approach advocated by many economists who have studied the question and spending very large amounts immediately on climate change mitigation to avoid this low probability possibility because they could be catastrophic. This approach assumes, of course, that what he has heard as to CAGW effects is of sufficient reliability to justify such early and overwhelming expenditures without careful analysis. As discussed in Section 2.6.2, however, application of the scientific method shows that the CSF is much less than assumed by the IPCC and may be in the range of 0.6 to 0.7 °C. This implies that CAGW effects are no more of a possibility now than they have been during the current Holocene period to date. But given the strong scientific case made in Section 2.3.1 above for low rather than high sensitivity, the scientific case for such outcomes disappears, and we are left with conventional benefit-cost analysis as a basis for making an economic decision.

Presumably given his earlier comments his views that CAGW effects are a realistic possibility are based on the global climate models used by the IPCC. So without examining the extent to which these climate models represent an accurate representation of the climate system, he appears to be proposing that very large amounts be spent starting immediately because of the output from these models. This appears to be a triumph of climate scare fears over careful physical science and economic analysis.

As discussed in Section 3 above, however, there exists an alternative approach to mitigation which would cost only a small fraction of the GHG emissions control approach, has a realistic possibility of actually controlling global climate change, and does not need to be implemented until actual CAGW effects should become imminent, if they ever do, since unlike CO_2_ emissions reductions the effects of SRM on global temperatures appear within months rather than decades. If indeed, Krugman is so concerned about the risks posed by such effects, he could have suggested a wait and see approach using these alternative approaches as a safety net, which is clearly the low cost approach to avoiding possible CAGW effects.

Although Krugman [65] refers to Weitzman (presumably [81]), Weitzman recognizes the possibility of using a geoengineering approach as a way to avoid the risks from less than likely CAGW effects. The principal costs are some early but much more affordable research to better understand which geoengineering approaches offer the best approaches, how it could best be implemented, and building an institutional structure that would facilitate its use [62].

More generally, Krugman takes an interest in the relevant science, but fails to ask the crucial questions concerning whether his scientific understanding corresponds with the most relevant observational data and the scientific method and whether there may be lower cost mitigation alternatives.

### 5.2. Lomborg

Lomborg [58] takes a somewhat different approach. He believes the climate models and that global warming will occur as they claim it will, but believes that there are other important problems that also need to be addressed in the world. He also has taken an interest in geoengineering and believes that it needs to be explored. So he is a moderate believer in the CAGW science, a strong believer that the costs of mitigation need to be reduced, primarily through a large tax-funded government research program, and that other alternatives need to be explored. So call him a moderate on the benefits of mitigation and a strong believer that costs can and need to be reduced if CO_2_ emissions are to be effectively reduced.

The multidisciplinary approach taken by Lomborg on the cost side is consistent with that advocated here, but it appears that Lomborg did not use and may not have sought the critical climate science inputs on the benefits side such as those described in Section 2 above. He may also have overestimated the effectiveness of large government research efforts that are not very clearly focused on a particular promising technology. Clearly the Manhattan Project was successful in reaching its goals, but the same cannot be said for governmental efforts to carry out research on climate change/global warming [60].

In sum, Lomborg does not appear to question the science advanced by climate alarmists but does examine whether there are lower cost mitigation alternatives.

### 5.3. Relation between Analysts’ Assumptions and Policy Recommendations

Although there have been some economics-only disagreements with regard to the economics of CAGW, principally with regard to the best discount rate to use and how to best value the benefits and costs involved, it appears that the physical science inputs used and other assumptions are at least as important in determining the policy recommendations arrived at. This article has emphasized the importance of the CSF and CO_2_ residence time in the atmosphere, which in turn determine the extent of the likely CAGW effects of global warming, the cost of improving energy efficiency, and whether geoengineering approaches are regarded as valid alternatives to reductions in GHG emissions as a mitigation approach.

What this suggests is the importance of carefully examining these physical science inputs and other assumptions in arriving at useful conclusions. It is only by doing this that the major variables in reaching an economic conclusion with regard to proposed climate change mitigation measures can be fully understood and explained. At the risk of gross generalizations from limited data, Table 4 shows my assessment of how many of the economic analysts treated the major assumptions and their overall policy recommendations. Obviously there are exceptions to the generalizations included in the table which are not noted, particularly in the last column given the diversity of the other studies. And there will be disagreements as to my characterizations. But I believe that the table suggests the major policy variables that have been considered to date. Economists might find it useful to give greater attention to the major physical science assumptions in preparing detailed benefit and cost estimates.

It appears that the conclusions from this analysis are substantially different from most previous such analyses for the reasons identified by this table. A major difference is the detailed review of some of the scientific and technical issues involved in this article.

## 6. Conclusions

### 6.1. Conclusions with Respect to the Economics of Climate Change Control

By taking a multidisciplinary approach carefully considering what other disciplines have to say, insisting on using the most relevant observational data and the scientific method rather than depending on assertions, climate model outputs, and questionable conclusions, and considering lower cost alternatives to achieving the same benefits leads to substantially different economic conclusions than most previous economic analyses, including the following:

- Athough there are significant uncertainties with regard to exactly what assumptions other economic analyses have made, a good case can be made that the economic benefits of reducing CO_2_ emissions may be about two orders of magnitude less than those previously estimated by most economists because the climate sensitivity factor (CSF) is much lower than assumed by the United Nations because feedback is negative rather than positive and the effects of CO_2_ emissions reductions on atmospheric CO_2_ are short rather than long lasting.
- The costs of CO_2_ emissions reductions are very much higher than usually estimated because of technological and implementation problems recently identified. Attempts to decrease these costs by a greatly expanded government funded research program to encourage technological innovation are both expensive and may or may not prove successful in reducing the technological problems.
- Geoengineering such as solar radiation management is a controversial alternative to CO_2_ emissions reductions that offers opportunities to greatly decrease these large costs, change global temperatures with far greater assurance of success, and eliminate the possibility of low probability, high consequence risks of rising temperatures, but has been largely ignored by economists. The costs of this promising but so far not carefully researched and validated approach appear to be many orders of magnitude cheaper, albeit with possible new and also little researched risks. It would, however, introduce the possibility of unforeseen new risks, but these risks could be reduced with relatively low cost research if carried out before any implementation. With such a modest research program a geoengineering option could provide an insurance policy against CAGW if that should ever become a realistic possibility. This approach should remove concern about low probability, high consequence events arising from increasing global temperatures since such SRM could be implemented extremely rapidly with adequate preparations, unlike (probably futile) attempts to reduce atmospheric CO_2_ levels decades in advance, as currently proposed by the UN and many Western governments. So there is no basis for taking any action currently to control climate change, but research on the implementation of geoengineering options such as SRM might be worthwhile.

### 6.2. More General Conclusions with Respect to Carrying Out Economic Analyses of Environmental Mitigation

Although it is not always necessary for environmental economists to understand the physical science aspects of the proposed environmental control measures proposed and to determine whether there may be lower cost means to achieve the benefits desired from the proposed mitigation, it certainly never does any harm and in most cases involving multidisciplinary issues it is vital if economists are to provide realistic and useful advice to decision makers. Some may object that in this specialized world economists should leave such matters to physical scientists since it is believed that they will know more about them. The danger, of course, is that economists may place their trust in physical scientists who are either not sufficiently knowledgeable or have a prior bias towards particular physical science hypotheses or mitigation methods to the exclusion of others. What is needed on the part of economists is an inquiring and open mind, insistence on use of the most relevant observational data and the scientific method, and technical curiosity so as to determine whether there may be lower cost or more efficient alternative methods to achieve whatever the environmental control measures they are evaluating are supposed to achieve. Economists do not have to carry out the physical science research involved or invent the lower cost control measures, but they do need to recognize which research and control measures meet their needs in these respects and particularly which have been validated by use of the most relevant observational data and the scientific method.

## Figures and Tables

**Table 1.** Correlation of various physical attributes with global temperatures. Source: d’Aleo [47].

**Factor**	**Years**	**Correlation (Pearson Coefficient)**	**Correlation Strength (R-squared)**
Carbon Dioxide (CO_2_)	1895–2007	0.66	0.43
Total Solar Irradiance (TSI)	1900–2004	0.76	0.57
Ocean Warming Index (PDO and AMO)	1900–2007	0.92	0.85
Carbon Dioxide Last Decade	1998–2007	–0.14	0.02

**Table 2.** Comparison of SRM and ERD approaches to climate mitigation^a^.

**Control Approach**	**Solar Radiation Management (SRM)**	**Exclusive Regulatory Decarbonization (ERD)**
Time to modify	Months	Decades at best
Ability to handle uncertainties	Very great	Very limited by need for new international negotiations
Catastrophic changes	Capable of fully avoiding if rapid action taken	50% probability at best of achieving less than 2 °C increase using IPCC assumptions^b^
Ocean acidification	No effect	Reduce w/difficulty, not solve^c^
Marginal cost/ton carbon equivalent	$0.02 to 0.10	$3,500 to achieve 2 °C w/50% probability^d^ assuming high IPCC CSF and long CO_2_ residence times in atmosphere
Cumulative overall costs Development Control	(undiscounted to 2100) ∼$0.001 × 10^12^ **∼**$0.090 × 10^12^	(undiscounted to 2100) ≫$0.45 × $10^12^^e^ ∼$2300 × 10^12^^f^
Effectiveness	Demonstrated by major volcanic eruptions to be very high	Probably fairly low given low CSF and unwillingness of humans to reduce GHGs
Other environmental effects	Unknown and untested but likely	Some already evident like rainforest destruction from oil palm expansion
Participation needed	Government involvement desirable initially; not required	Mandatory actions by most governments, companies, and people

Sources:

a. Based on Carlin [55], Table 1, which is based on Carlin [60], unless otherwise stated. IPCC assumptions used for ERD.

b. Rive, *et al*. [56], Table 1. This assumes a goal of staying below a 2 °C temperature increase from pre-industrial levels in order to avoid dangerous climate changes as per European Union policy.

c. See discussion in [55], Section II.B.3, pp. 734–735.

d. Rive *et al*. [56], p. 385.

e. Nova [59], p. 17 shows about $5 billion per year in the last few years for climate science and technology expenditures for the US alone. Other nations, particularly in Western Europe, have also had substantial expenditures. These expenditures have substantially increased under the Obama Administration, as shown in the Nova 2009 total, and would be very likely to be much higher if serious CO_2_ emission reductions should be undertaken.

f. Galiana and Green [57], p. 20.

**Table 3.** Some previous estimates of the economic benefits of climate change control.

**Study**	**Warming (°C)**	**Impact (%GDP)**
Nordhaus (1994) [66]	3.0	−1.3
Nordhaus (1994) [67]	3.0	–4.8 (–30 to 0)
Fankhauser (1995) [68]	2.5	−1.4
Tol (1995) [69]	2.5	−1.9
Nordhaus and Yang^a^ (1996) [70]	2.5	−1.7
Plamberk and Hope^a^ (1996) [71]	2.5	−2.5 (–0.5 to −11.4)
Mendelsohn *et al*.^a,b,c^ (2000) [72]	2.5	0.0^b^
		0.1^b^
Nordhaus and Boyer (2000) [73]	2.5	−1.5
Tol (2002a) [74]	1.0	2.3
Maddison (2003) ^a,d,e^[75]	2.5	−0.1
Rehdanz and Maddison^a,c^ (2005) [76]	1.0	−0.4
Hope (2006) ^a,f^ [77]	2.5	0.9 (–0.2 to 2.7)
Nordhaus (2006) [78]	2.5	−0.9 (0.1)
Stern (2006) [63]		−5 to as much as −20%
Garnaut (2008) [64]	5.1	
Krugman (2010) [65]	(5.0) ^g^	(–5) ^g^

Sources: Tol [54] summary for first 13 studies. Fragmentary data for last three based on this author’s reading of these studies. Notes on Table:

a. The global results were aggregated by Tol [54].

b. The top estimate is for the “experimental” model, the bottom estimate for the “cross-sectional” model.

c. Mendelsohn *et al*. only include market impacts.

d. The national results were aggregated to regions by the current author for reasons of comparability.

e. Maddison only considers market impacts on households.

f. The numbers used by Hope are averages of previous estimates by Fankhauser and Tol; Stern *et al*. (2006) [63] adopted the work of Hope.

g. Krugman does not explicitly endorse these figures but rather speaks highly of them on pdf page 8.

**Table 4.** Comparison of principal assumptions and recommendations by economic analysts.

**Assumption/Analyst**	**Krugman [65]**	**Lomborg [58]**	**Government Reports [63,64]**	**Carlin**	**Most Others [66–78]**
Ultra-low discount rate	No	No	Yes (0.1% [63], 0.05% [64])	No	No
Optimistic technology costs	Assumes low costs—so yes	No	Yes	No (Sec. 3.2)	Yes
Energy efficiency research effective	Not discussed	Yes	Yes	No (Sec. 3.2)	Not discussed
Catastrophic threat high	Yes	No	Presumably	No (Sec. 2.5)	Varies
High CSF	Presumably	Yes	Yes	No (Sec. 2.3)	Yes
CO_2_ residence time in atmosphere	Presumably long	Presumably long	Presumably long	Short (Sec. 2.2 & 2.6.1)	Presumably long
Critical examination of scientific validity	No	No	No	Yes (Sec. 2)	No
Geoengineering valid alternative	Not discussed	Yes	Not discussed	Yes (Sec. 3.3)	Not discussed
Principal policy recommendation and basis	“Big bang” to reduce threat of CAGW	Energy efficiency research to reduce costs	“Big bang” to avoid “dangerous” CO_2_ levels	No action; geoengineering research (Sec. 3.3 & 4)	“Policy ramp” to reduce discounted costs

## List of Acronyms

AMO: Atlantic Multidecadal Oscillation
AR4: Fourth Assessment Report of the IPCC published in 2007
CAGW: Catastrophic AnthropogenicGlobal Warming
CO_2_: Carbon Dioxide
CSF: Climate Sensitivity Factor
°C: Degrees Centigrade
δ^13^C: Isotopic ratio of ^13^C to ^12^C defined as the standard-normalized difference from the Pee Dee Belemnite (PDB) standard
ENSO: El Niño Southern Oscillation
ERD: Exclusive Regulatory Decarbonization
EU: European Union
GCM: General Circulation Model
GDP: Gross Domestic Product
GWP: Global World Output
GHG: Greenhouse Gas
IPCC: (UN) Intergovernmental Panel on Climate Change
MER: Market Exchange Rates
PDO: Pacific Decadal Oscillation
RCIO: Carbon Intensity of Output
RT: Residence Time
SRM: Solar Radiation Management
TSI: Total Solar Irradiance
US: United States
US$: United States dollar
UN: United Nations
